# Ameliorative Effects of Ethyl-Acetate Extract of *Bidens pilosa* on Oxidative Stress and Reproductive Impairment in Bisphenol A-Exposed Male Mice: Insight from In-silico, Invitro and In-vivo Studies

**DOI:** 10.12688/f1000research.166336.2

**Published:** 2025-12-02

**Authors:** Ismahil Adekunle Adeniyi, Daniel Owu, Olufunke Onaadepo, Umar Uthman Shehu, Ishak Abdi Jama, Joshua Ojodale Aruwa, Theophilus Pius, Ibe Michael Usman

**Affiliations:** 1 Department of Physiology, Faculty of Biomedical Sciences, Kampala International University - Western Campus, Bushenyi, Western Region, Uganda; 2 Department of Anatomy, Faculty of Biomedical Sciences, Kampala International University - Western Campus, Bushenyi, Western Region, Uganda; 3 Department of Pharmacology & Toxicology, School of Pharmacy, Kampala International University - Western Campus, Bushenyi, Western Region, Uganda; 4 Department of Medical Laboratory Science, Kampala International University - Western Campus, Bushenyi, Western Region, Uganda

**Keywords:** Bidens pilosa, Bisphenol A, Antioxidant, Oxidative stress, Reproductive toxicity.

## Abstract

**Background:**

Bisphenol A (BPA), a ubiquitous environmental pollutant, is known to induce oxidative stress and disrupt male reproductive function. This study evaluated the ameliorative effects of the ethyl-acetate extract of *Bidens pilosa L., Asteraceae* (EABP) on oxidative stress and reproductive impairment in BPA-exposed male mice.

**Method:**

GC-MS phytocompounds from EABP were subjected to *in-silico* pharmacokinetic, drug-likeness, and toxicological screening using the SwissAdme and PkCSM web servers. Antioxidant capacity of EABP was determined using 2,2-diphenyl-1-picrylhydrazyl (DPPH) radical scavenging assay. Over a 28-day period, male mice were assigned to six groups. The control group (Group 1) received distilled water, while Group 2 administered BPA at a dose of 100 mg/kg/day. Groups 3, 4, and 5 were treated with BPA in combination with EABP at doses of 250, 500, and 1000 mg/kg/day, respectively. Group 6 received BPA along with vitamin C at 100 mg/kg/day. Various parameters, including body weight, malondialdehyde, catalase, and superoxide dismutase level were assessed.

**Result:**

Molecular docking analysis revealed that 9,12,15-octadecatrienoic acid, a key phytoconstituent of EABP, exhibited strong binding affinity for both the androgen receptor (AR) and nuclear factor erythroid 2-related factor 2 (NRF2). Its interaction with AR was comparable to that of testosterone, suggesting potential androgenic activity, while its binding to NRF2 mirrored that of vitamin C. The extract’s antioxidant activity was further supported by the DPPH radical scavenging assay, where it demonstrated moderate potency with an IC₅₀ of 6.11μg/mL, compared to 1.25μg/mL for vitamin C. in vivo findings showed that treatment with EABP at 250 mg/kg, 500 mg/kg, and 1000 mg/kg restored SOD activity and significantly reduced MDA levels. Catalase (CAT) activity also improved, particularly at the higher dose.

**Conclusion:**

Collectively, these results indicate that the EABP offers protective effects against BPA-induced oxidative stress, metabolic disturbance, and reproductive dysfunction, possibly through its combined antioxidant and hormone-like properties.

## Introduction

The increasing prevalence of male infertility worldwide has raised significant concern among reproductive health researchers. ^1^ Among the many environmental factors implicated, oxidative stress—an imbalance between the generation of reactive oxygen species (ROS) and the antioxidant defense system—plays a pivotal role in impairing testicular function and spermatogenesis. ^2^ One of the environmental pollutants strongly associated with reproductive dysfunction is Bisphenol A (BPA), an industrial chemical commonly found in polycarbonate plastics and epoxy resins. ^3^ BPA mimics estrogen and disrupts the endocrine system, leading to altered hormone levels, testicular damage, and poor semen quality. ^4^ Importantly, BPA exerts its toxic effects largely through the induction of oxidative stress, which results in lipid peroxidation, protein oxidation, DNA damage, and apoptosis in reproductive tissues.

Several biomarkers are used to assess oxidative damage and antioxidant responses in biological systems. Malondialdehyde (MDA) is a by-product of lipid peroxidation and serves as a reliable indicator of cellular membrane damage. ^5^ On the other hand, enzymatic antioxidants such as superoxide dismutase (SOD) and catalase (CAT) play essential roles in neutralizing ROS. ^6^ SOD catalyzes the conversion of superoxide radicals to hydrogen peroxide, while CAT breaks down hydrogen peroxide into water and oxygen. A decline in the activity of these enzymes typically signifies compromised antioxidant defense, as seen in BPA-induced reproductive toxicity. ^7^

Amid growing concerns over synthetic antioxidant drugs and their side effects, attention has shifted toward plant-derived compounds with potential therapeutic benefits. *Bidens pilosa L., Asteraceae*, a tropical and subtropical plant widely used in traditional medicine, has attracted scientific interest due to its rich phytochemical profile, including flavonoids, phenolic acids, terpenoids, and alkaloids. ^8^ These bioactive compounds are believed to exert significant antioxidant, anti-inflammatory, and cytoprotective effects. ^9^ Of particular interest is the ethyl-acetate fraction of *B. pilosa*, which selectively concentrates lipophilic and semi-polar compounds with potent biological activity.

To evaluate the antioxidant potential of *B. pilosa* against BPA-induced reproductive oxidative stress, this study employed both in vitro and in vivo approaches. in vitro analysis involved the 2,2-diphenyl-1-picrylhydrazyl (DPPH) radical scavenging assay—a standard method used to determine the free radical-scavenging capacity of plant extracts. In vivo, the extract’s efficacy was assessed by measuring changes in SOD and CAT activities and MDA levels in testicular tissues of BPA-exposed male mice, serving as indicators of oxidative balance and lipid peroxidation.

Moreover, to elucidate the possible molecular interactions between key phytochemicals in *B. pilosa* and biological antioxidant targets, molecular docking studies were conducted. This computational approach simulates the binding affinity and orientation of plant-derived compounds with proteins involved in oxidative stress pathways, providing mechanistic insights that complement the experimental findings. By identifying specific ligand-protein interactions, molecular docking helps to predict which constituents of the extract may directly modulate oxidative defense mechanisms at the molecular level.

In summary, this study investigates the protective effects of the ethyl-acetate extract of *Bidens pilosa* against BPA-induced oxidative stress in the male reproductive system. Through a combination of DPPH-based radical scavenging assays, biochemical evaluation of SOD, CAT, and MDA, and molecular docking analysis, the study aims to validate the antioxidant efficacy of *B. pilosa* and explore its potential as a natural remedy for environmental toxin-induced male infertility.

## Methodology

### Ethical clearance

Prior to the commencement of this study, ethical approval was obtained from the Research Ethics Committee of Kampala International University, Western Campus (Approval No. KIU-2024-532). In addition, authorization to conduct the study was granted by the Uganda National Council for Science and Technology (UNCST), under approval number HS5372ES.

### Chemicals

Bisphenol A (Cat. No: 239658) was used to induce endocrine disruption and systemic toxicity in experimental animals at a dose of 100 mg/kg body weight administered orally once daily. Ethyl acetate (Cat. No: 270989) was employed as a solvent during the extraction and fractionation of plant materials, with approximately 100 mL used per extraction batch. Methanol (Cat. No: 34860) was used both as a solvent in the DPPH radical scavenging assay and for plant extraction procedures, with an estimated volume of 100 mL per assay and extraction cycle. DPPH (Cat. No: D9132) powder (0.004% w/v in methanol) was used to evaluate the antioxidant activity of the plant extracts through a radical scavenging assay. Ketamine (Cat. No: PHR1663) and Xylazine (Cat. No: X1251) were used in combination as anesthetics prior to animal sacrifice, administered intraperitoneally at doses of 80 mg/kg and 10 mg/kg body weight, respectively. Eosin-nigrosin (Cat. No: 134-25-8/N3268) stain was used for assessing sperm viability and membrane integrity, with one drop of each stain applied to a semen smear for microscopy. They were purchased from Sigma-Aldrich (St. Louis, MO, USA). All other reagents used in the study were of analytical grade and obtained from Reagent World Ltd. (Kampala, Uganda), an authorized distributor of Sigma-Aldrich products.

### Plant extraction

Fresh leaves of *Bidens pilosa* were collected in September 2024 from the vicinity of Ishaka Mosque, located in the Ishaka-Bushenyi District of Uganda. Botanical authentication was conducted by Dr Olet Eunice of the Department of Botany at Mbarara University of Science and Technology, where a voucher specimen (No. AAI-2024-001) was deposited in the departmental herbarium. The collected leaves were thoroughly air-dried in a shaded area to prevent photodegradation for five (5) days and then ground into a fine powder using a blending machine (Model XYZ123, Philips, Amsterdam, Netherlands).

A portion of the powdered material (100 g) was first filtered to remove coarse plant residues and then suspended in 400 mL of distilled water for 48 hours with intermittent shaking. The aqueous suspension was subsequently partitioned successively with solvents of increasing polarity using a separatory funnel. The solvents used for fractionation included *n*-hexane, dichloromethane, ethyl-acetate, and *n*-butanol. Each solvent was added, mixed thoroughly with the aqueous layer, and the resulting organic phase was collected following phase separation. This process was repeated three times per solvent to ensure exhaustive extraction.

Each solvent fraction— *n*-hexane, dichloromethane, ethyl-acetate, and *n*-butanol—was individually concentrated under reduced pressure using a rotary evaporator to obtain the respective crude fractions. The concentrated extracts were stored at 4 °C until further use for phytochemical screening and biological assays.

### Chemical characterization and molecular docking

Phytochemical constituents in the crude extracts were identified using Gas Chromatography-Mass Spectrometry (GC-MS) analysis, performed on a Shimadzu QP2010 GC-MS system equipped with an RTX-5MS capillary column (30 m × 0.25 mm ID × 0.25 μm film thickness). Helium served as the carrier gas at a constant flow rate of 1.0 mL/min. The injector temperature was maintained at 250 °C. The oven temperature program began at 60 °C (held for 2 minutes), ramped at 10°C/min to 280 °C, and was then held isothermally for 10 minutes.

The mass spectrometer operated in electron ionization (EI) mode at 70 eV with a scan range of 40–600 m/z. Compound identification was performed by comparing the acquired mass spectra against entries in the National Institute of Standards and Technology (NIST) library database, along with comparisons of retention indices and fragmentation patterns to those of authentic reference standards where available. Chromatographic peaks corresponding to individual compounds were quantified based on peak area and height. Identified compounds were characterized by their chemical names, molecular weights, and molecular formulas, as determined through spectral matching and library searches.

The bioactive compounds identified through GC-MS analysis were further evaluated in silico to predict their physicochemical properties, drug-likeness, lipophilicity, and solubility profiles. These predictions were based on established medicinal chemistry filters, including Lipinski’s Rule of Five, the Ghose filter, Veber rule, and Egan rule, as implemented in the SwissADME web tool. ^10^ Additionally, pharmacokinetic parameters such as gastrointestinal (GI) absorption, P-glycoprotein (P-gp) substrate potential, cytochrome P450 (CYP) enzyme inhibition (focusing on CYP1A2 and CYP2D6), and skin permeability (log Kp) were also predicted using the same platform.

To gain insights into the possible biological activities of the compounds, molecular docking simulations were carried out using PyRx software, employing the AutoDock Vina algorithm. ^11^ Docking studies were focused on two key protein targets—Nuclear factor erythroid 2-related factor 2 (Nrf2) and the Androgen Receptor (AR)—due to their central roles in oxidative stress regulation and male reproductive function, respectively.

High-resolution (≤2.5 Å) three-dimensional structures of Nrf2 and AR were retrieved from the Protein Data Bank (PDB). Preference was given to structures co-crystallized with ligands to aid in accurate definition of the active site. Protein preparation was performed using AutoDock Tools (ADT) v1.5.7 and UCSF Chimera v1.15. This involved the removal of water molecules, co-crystallized ligands, and heteroatoms, addition of polar hydrogens and Kollman charges, atom type assignment, and conversion to the PDBQT format required for docking. Energy minimization of the protein structures was carried out in Chimera using the AMBER ff14SB force field to relieve structural strain and enhance docking accuracy.

Compounds identified in *Bidens pilosa* through GC-MS analysis—namely Methyl (Z)-5,11,14,17-eicosatetraenoate, Pentadecanoic acid, Phytol, 9,12,15-Octadecatrienoic acid, Squalene, and Hexatriacontane—were selected for further molecular docking based on a relative abundance threshold of ≥2% peak area. Ligand structures were retrieved from the PubChem database in SDF format and subsequently converted to 3D conformations using Open Babel, which also handled format conversion and protonation state adjustment. Geometry optimization was performed using Avogadro software with the MMFF94 force field.

Ligands were parameterized using the General AMBER Force Field (GAFF) through ACPYPE or Antechamber to generate MOL2 and PDBQT files compatible with docking software. Among the ligands, 9,12,15-Octadecatrienoic acid, which exhibited the highest binding affinity in preliminary docking, was selected for further interaction analysis.

Molecular docking was conducted using AutoDock Vina v1.2.3, chosen for its accuracy and computational efficiency. The docking grid was centered on the active site of the target proteins, defined by known ligand-binding residues, and sized to accommodate ligand flexibility. Docking results were ranked according to binding energy scores (kcal/mol), with the top-ranked poses retained for post-docking analysis.

Visualization and interaction analysis were performed using multiple tools: Discovery Studio Visualizer (BIOVIA) was employed to examine hydrogen bonding, hydrophobic interactions, and molecular surface characteristics; PyMOL was used for high-resolution 3D visualization and figure preparation; and LigPlot+ was utilized to generate 2D interaction diagrams highlighting key amino acid contacts involved in ligand binding.

### DPPH radical scavenging assay

In this study, DPPH and ascorbic acid (used as a standard antioxidant) were procured from a certified supplier and used without further modification. Methanol of analytical grade served as the solvent for all preparations. Serial dilutions of the ethyl acetate extract of *Bidens pilosa* were prepared at concentrations of 20, 30, 40, and 50 μg/μL, while ascorbic acid solutions were prepared at concentrations of 0.1, 0.2, 0.3, and 0.4 μg/μL. A 0.1 mM DPPH solution was freshly prepared in methanol. For each reaction, 1 mL of the test sample or standard solution was mixed with 1 mL of the DPPH solution. The control consisted of 1 mL methanol mixed with 1 mL DPPH solution.

All mixtures were incubated in the dark at room temperature for 30 minutes to prevent photodegradation. Following incubation, absorbance readings were taken at 517 nm using a UV-Visible spectrophotometer (UV-1800, Shimadzu Corporation, Kyoto, Japan). The percentage of radical scavenging activity was calculated relative to the control.

The percentage of DPPH radical scavenging activity was calculated using the formula:

$$\%\text{Inhibition}=\frac{A\;\text{control}-A\;\text{sample}}{A\;\text{control}}\times 100$$

Where A control is the absorbance of the control and A sample is the absorbance of the test sample.

A standard curve was plotted for ascorbic acid to determine the IC _50_ (the concentration required to inhibit 50% of DPPH radicals). Similarly, the IC _50_ for the plant extract was calculated from its linear regression equation.

IC _50_ stands for “Half Maximal Inhibitory Concentration.”

IC _50_ is the amount of a substance needed to reduce an activity (like radical activity) by half. It’s commonly used to measure the potency of an antioxidant, drug, or inhibitor—the lower the IC _50_, the more potent the substance.

### Acute toxicity

The up-and-down technique for acute toxicity was used to determine the LD50 of the methanol extract of *Bidens pilosa* as outlined by. ^12^ This technique provides accurate information on the extract’s toxicological profile while minimizing the usage of animals. At the end of this procedure 5 mice were used for the acute toxicity study. The oral LD50 of ethyl-acetate extract of *Bidens pilosa* was found to be greater than 5000 mg/kg body weight suggesting that the plant is safe.

### Experimental animals

Thirty inbred adult male albino mice ( *Mus musculus*), each weighing between 35–45 g, were used for this study. The animals were sourced from the Animal House of Mbarara University of Science and Technology, Uganda. Upon procurement, the mice were transported to the Animal House of Kampala International University, Western Campus, where they were housed in clean, well-ventilated plastic cages (five mice per cage) and acclimatized for a period of two weeks prior to the commencement of experimental procedures. During acclimatization and throughout the study, the animals were maintained under standard laboratory conditions: a controlled room temperature of 22 ± 2°C, relative humidity of 70 ± 4%, and an inverted 12-hour light/dark cycle. The mice were given unrestricted access to standard pelleted rodent feed and clean drinking water ad libitum. Bedding material (wood shavings) was changed every two days to maintain hygiene.

All procedures involving animals were conducted in accordance with the guidelines of the Institutional Animal Care and Use Committee (IACUC, 2010) and adhered to the UK Animals (Scientific Procedures) Act 1986, as amended in 2012. The ethical approval for this study was obtained from the Kampala International University Institutional Research and Ethics Committee (KIU-REC).

### Sample size justification

The sample size was determined using the power resource equation;

Minimumn=10/k+1,Maximumn=20/k+1

where n is the number of animals per group, k is the number of groups. ^13^

Therefore, the minimum number of animals needed per group for the studies = (10/6) +1 = 2.7.

While the maximum number of animals needed per group for the studies = (20/6) +1 = 4.3.

Hence, we 5 animals were assigned to the 6 groups.

### Experimental designs

After 2 weeks of acclimatization, the mice were randomly assigned into six groups with five mice in each group to reduce bias in accordance with ARRIVE guidelines. ^14^

Group 1 (control) received 2 ml/kg body weight of distilled water; Group 2 (100 mg/kg/day of BPA) were administered only bisphenol A; Group 3 (100 mg/kg/day of BPA + 250 mg/kg BW extract of *B. pilosa*) received bisphenol A and co-treated with 250 mg/kg body weight of the leaf extract; Group 4 (100 mg/kg/day of BPA + 500 mg/kg BW extract of *B. pilosa*) received BPA and co-treated with 500 mg/kg body weight of leaf extract; Group 5 (100 mg/kg/day of BPA + 1000 mg/kg BW extract of *B. pilosa*) received BPA and co-treated with 1,000 mg/kg of leaf extract, while Group 6 (100 mg/kg/day of BPA + Vit. C 60 mg/kg BW) was administered BPA and vitamin C. All treatments were administered once daily via oral gavage for a period of 35 consecutive days. BPA was freshly prepared and administered each day at a consistent time in the morning to minimize circadian variation. The dosage for BPA (100 mg/kg/day) was adopted from a previous study by. ^15^ The dosage for *B. pilosa* (250 mg/kg, 500 mg/kg, and 1000 mg/kg) was adopted from a previous study by, ^16^ while that of Vitamin C was also adopted from previous study by. ^17^ The body weights of all animals in each group were recorded weekly, beginning from the first week of the experiment till the end of the administration period at the fifth week. At the end of the treatment period, all animals were fasted overnight and weighed prior to euthanasia procedures. Anesthesia was induced via intraperitoneal injection using a combination of ketamine hydrochloride at a dose of 80 mg/kg and xylazine hydrochloride at 8 mg/kg body weight. Adequate anesthesia was confirmed by the absence of a pedal reflex and other responses to external stimuli. Following confirmation of a deep plane of anesthesia, euthanasia was performed via exsanguination by cardiac puncture, consistent with the guidelines provided in the American Veterinary Medical Association (AVMA) Guidelines for the Euthanasia of Animals (2020 Edition). These procedures were carried out to minimize pain and distress and complied fully with institutional and international standards for the humane treatment of laboratory animals. Following confirmation of death by cessation of heartbeat and respiratory movement, the animals were dissected, and relevant tissues were harvested for histological and biochemical analyses.

### Oxidative stress markers and antioxidant enzyme determination

The testes were homogenized in Tris buffer using a mortar and pestle to prepare a 10% (w/v) tissue homogenate. The homogenate was then centrifuged at 3,000 × g for 10 minutes, and the resulting supernatant was collected for subsequent biochemical analyses. The supernatant was used to measure malondialdehyde (MDA) levels, superoxide dismutase (SOD) activity, and catalase activity.

MDA levels, an indicator of lipid peroxidation, were assessed using the thiobarbituric acid reactive substances (TBARS) assay, in which MDA reacts with thiobarbituric acid to form a pink chromogen detectable spectrophotometrically at 532 nm.

SOD activity was determined by its ability to inhibit the autoxidation of epinephrine at pH 10.2, as described by, ^18^ and was expressed as units per mg of protein. Catalase activity was measured according to the method of, ^19^ by monitoring the decomposition of hydrogen peroxide at 240 nm, with activity expressed as units per mg of protein.

### Statistical analysis

The data from the in vivo study were analyzed using GraphPad Prism ^®^ version 5.01 (San Diego, CA, USA). One-way analysis of variance (ANOVA) was used to assess the difference mean among the different groups; this was followed by Tukey’s post-hoc test, where necessary. Differences among the groups were considered significant where the p-value was equal or less than 0.05. The data for the study can be accessed as an extended file on figshare. ^14^

## Result

### Characterization of ethyl-acetate extracts of *Bidens pilosa* by GC-MS analysis

**Figure 1.**
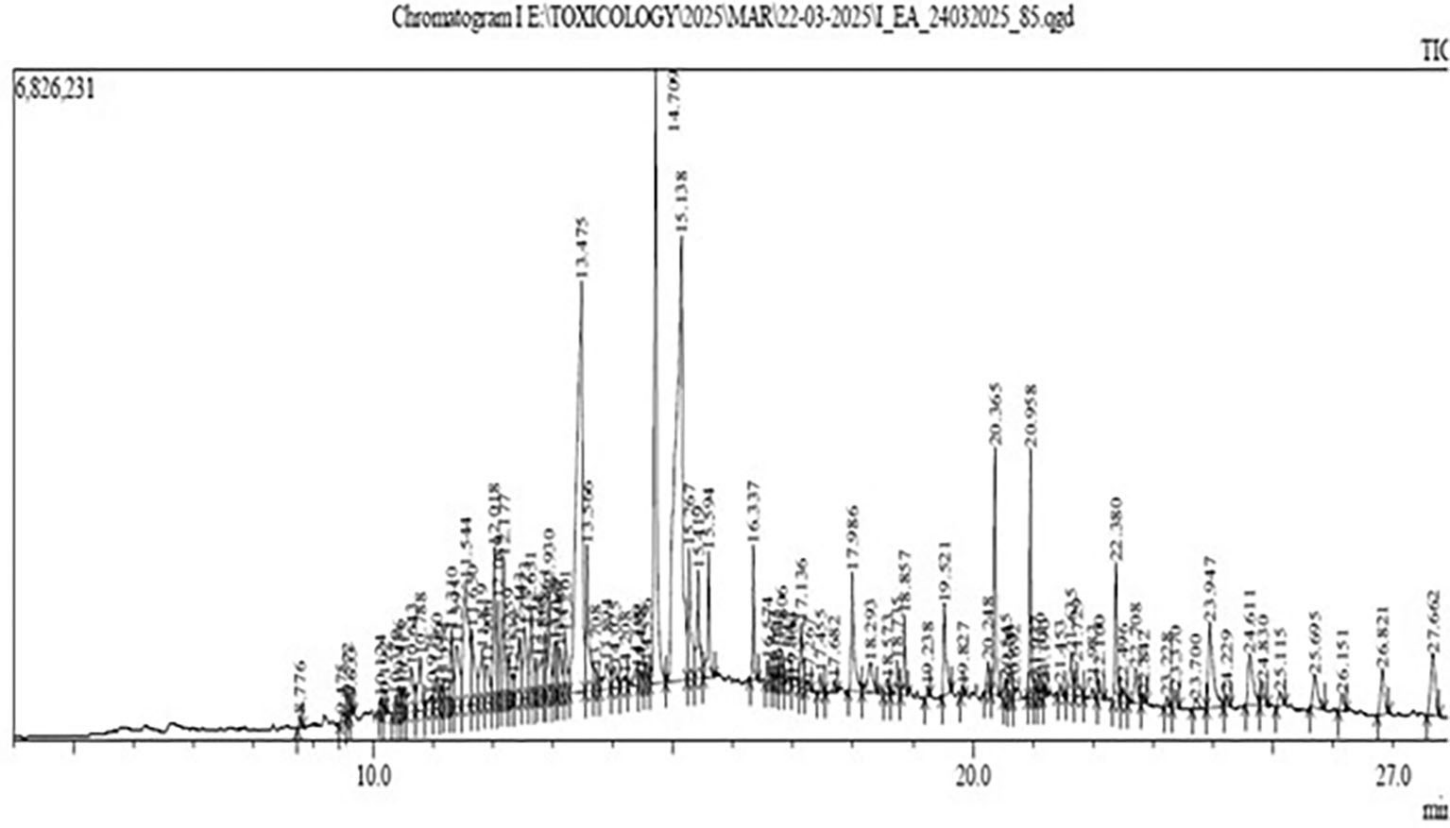
Result of GC-MS analysis of ethyl-acetate extract of *B. pilosa* revealing several constituents with different retention time and area percentage.

Gas Chromatography-Mass Spectrometry (GC-MS) analysis of the ethyl-acetate extract of Bidens pilosa revealed several major constituents as shown in Figure 1.

A higher area percentage in GC-MS analysis reflects the relative abundance and potential biological relevance of the detected compounds. Based on a minimum threshold of 2% area, six major compounds were identified for further characterization and analysis. These included Methyl (Z)-5,11,14,17-eicosatetraenoate, Pentadecanoic acid, Phytol, 9,12,15-Octadecatrienoic acid, Squalene, and Hexatriacontane ( Table 1). Compound identification was achieved by comparing the obtained mass spectra with reference spectra from established databases, considering key parameters such as retention time, molecular weight, molecular formula, and area percentage. The identities and bioactivity profiles of the selected compounds ( Table 2) were further validated using online chemical databases including PubChem, SwissADME, and ADMETlab 3.0, which collectively provide structural, pharmacokinetic, and physicochemical data for over 60,000 compounds.

**Table 1.** Phytochemical constituents identified in the ethyl-acetate extract of *Bidens pilosa* using Gas Chromatography-Mass Spectrometry (GC-MS).

Compound peak number	Compound name	Retention time	Area percentage (%)	Molecular weight (g/mol)	Molecular formula
18	Methyl (Z)-5,11,14,17-eicosatetraenoate	11.544	3.27	318.5	C _21_H _34_O _2_
38	Pentadecanoic acid	13.475	11.93	242.4	C _15_H _30_O _2_
47	Phytol	14.709	8.02	296.5g	C _20_H _40_O
48	9,12,15-Octadecatrienoic acid	15.138	15.20	278.4	C _18_H _30_O _2_
72	Squalene	20.365	2.47	410.7	C _30_H _50_
76	Hexatriacontane	20.958	2.31	507	C _36_H _74_

**Table 2.** Physicochemical properties, lipophilicity, and solubility of the compounds.

S/N	Compound	SMILES	MF	MW	WLogP	(Å ^2^)	HBD	HBA	Log S	SC
1.	Methyl (Z)-5,11,14,17-eicosatetraenoate	CC/C=C\C/C=C\C/C=C\CCCC/C=C\CCCC(=O) OC	C _21_H _34_O _2_	318.5 g/mol	6.31	26.30	0.0	2.0	-6.217	
2.	Pentadecanoic acid	CCCCCCCCCCCCCCC(=O) O	C _15_H _30_O _2_	242.4 g/mol	5.16	37.30	1.0	2	-4.66	Moderately soluble
3.	Phytol	C [C@@H] (CCC [C@@H](C)CCC/C(=C/CO)/C)CCCC(C) C	C _20_H _40_O	296.5 g/mol	6.36	20.23	1	1	-5.98	Moderately soluble
4.	9,12,15-Octadecatrienoic acid	CCC=CCC=CCC=CCCCCCCCC(=O)O	C _18_H _30_O _2_	278.4 g/mol	5.66	37.30	1	2	-4.78	Moderately soluble
5	Squalene	CC(=CCC/C(=C/CC/C(=C/CC/C=C(/CC/C=C(/CCC=C(C)C)\C)\C)/C)/C) C	C _30_H _50_	410.7 g/mol	10.60	0.00	0	0	-10.531	
6	Hexatriacontane	CCCCCCCCCCCCCCCCCCCCCCCCCCCCCCCCCCCC	C _36_H _74_	507 g/mol	14.29	0.00	0	0	-12.85	Insoluble

MF = Molecular Formula; MW = Molecular Weight in g/mol; logP = Lipophilicity measured by the atomistic method; Å ^2 ^= Topological Polar Surface Area (TPSA); HBD = Number of H-bond donor; HBA = Number of H-bond acceptors; RB = Number of rotatable bonds; Log S = Estimated water solubility value by topological method; SC = Solubility class based on the Log S value.

### Pharmacokinetics analysis of identified compounds

Of the six identified compounds, only two were predicted to have high gastrointestinal (GI) absorption, while the remaining four exhibited low predicted absorption. Among them, Phytol was uniquely identified as a substrate of P-glycoprotein (P-gp). None of the compounds were predicted to inhibit cytochrome P450 2D6 (CYP2D6); however, Pentadecanoic acid and 9,12,15-Octadecatrienoic acid were predicted to inhibit CYP1A2. The predicted skin permeability (log Kp) values for the compounds ranged from 0 to 4.18 cm/s, as summarized in Table 3.

**Table 3.** Pharmacokinetics properties and drug-likeness of the compounds.

S/N	Compound	GI absorption	Drug likeness	P-gp substrate	CYP1A2 inhibitor	CYP2D6 inhibitor	Log *K*_p_ cm/s
1.	Methyl (Z)-5,11,14,17-eicosatetraenoate	Low	Yes		No	No	
2.	Pentadecanoic acid	High	Yes Bioavailability score: 0.85	No	Yes	No	-3.07
3.	Phytol	Low	Yes Bioavailability score: 0.55	Yes	No	No	-2.29
4.	9,12,15-Octadecatrienoic acid	High	Yes Bioavailability score: 0.85	No	Yes	No	-3.41
5.	Squalene	Low		No	No	No	0
6.	Hexatriacontane	Low	No Bioavailability score: 0.17	No	No	No	4.18

GI = gastrointestinal; P-gp = P-glycoprotein; Log *K*_p_ = skin permeation (cm/s).

P-glycoprotein (P-gp) is a key transmembrane efflux transporter involved in the active removal of a wide range of xenobiotics from cells, playing a critical role in drug absorption, distribution, and excretion. In parallel, the cytochrome P450 enzyme family, particularly CYP2D6 and CYP1A2, is integral to drug metabolism. CYP2D6 metabolizes a diverse array of drugs, including antidepressants, antipsychotics, antiarrhythmics, and opioids. ^20^ Inhibition of CYP2D6 can impair drug metabolism, leading to elevated plasma concentrations and an increased risk of toxicity. ^21^ Similarly, CYP1A2 is responsible for metabolizing various drugs and endogenous substances. Its inhibition may reduce clearance rates, thereby altering drug pharmacokinetics and therapeutic efficacy. ^22^ Additionally, transdermal drug delivery is influenced by skin permeability, which is determined by factors such as molecular weight, lipophilicity, and skin condition. Log Kp values are commonly used to estimate a compound’s ability to penetrate the skin barrier.

### Molecular docking and docking analysis of compounds

**Figure 2.**
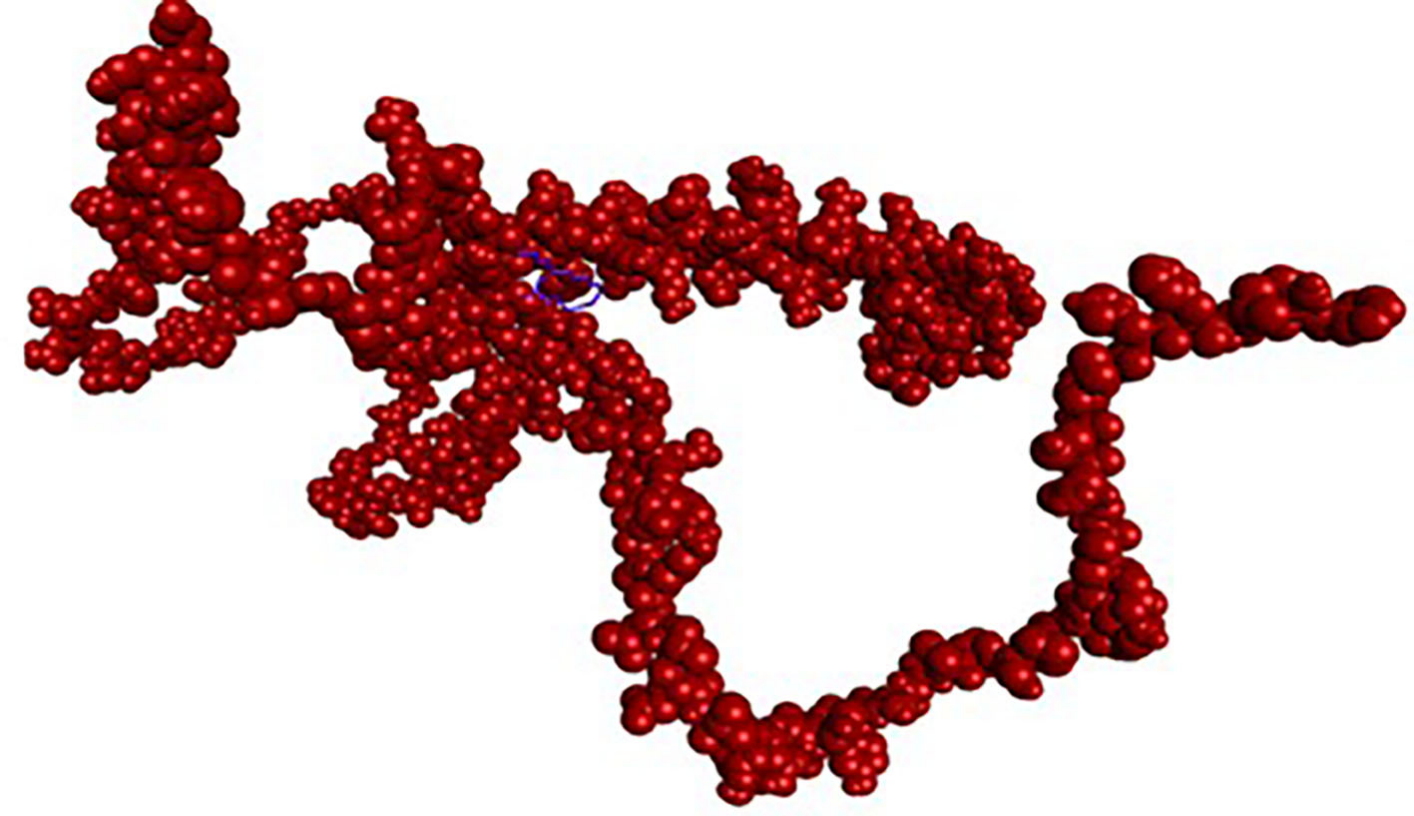
NRF2 protein and its binding site.

**Figure 3.**
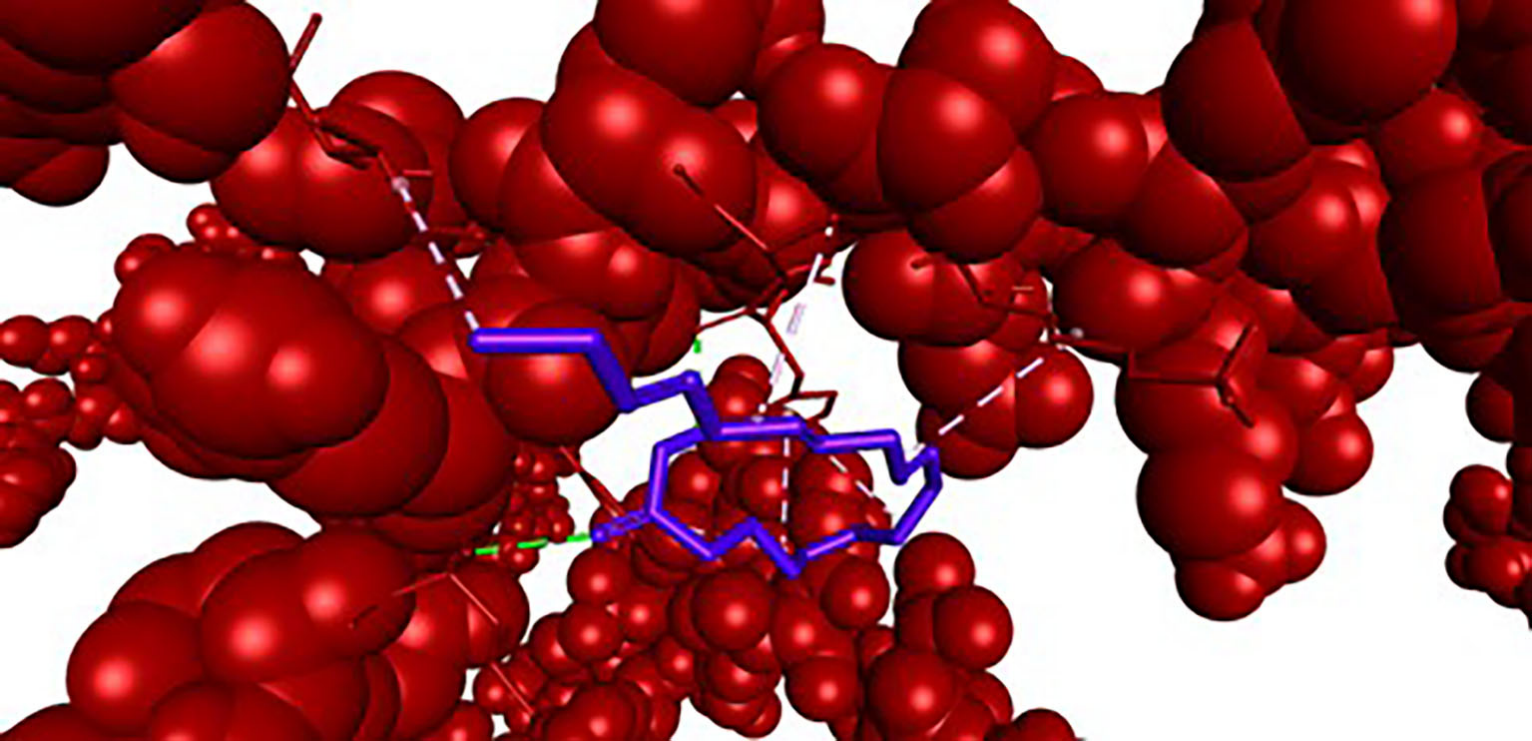
Closer view interaction between NRF2 protein and ligand (9,12,15-Octadecatrienoic acid).

**Figure 4.**
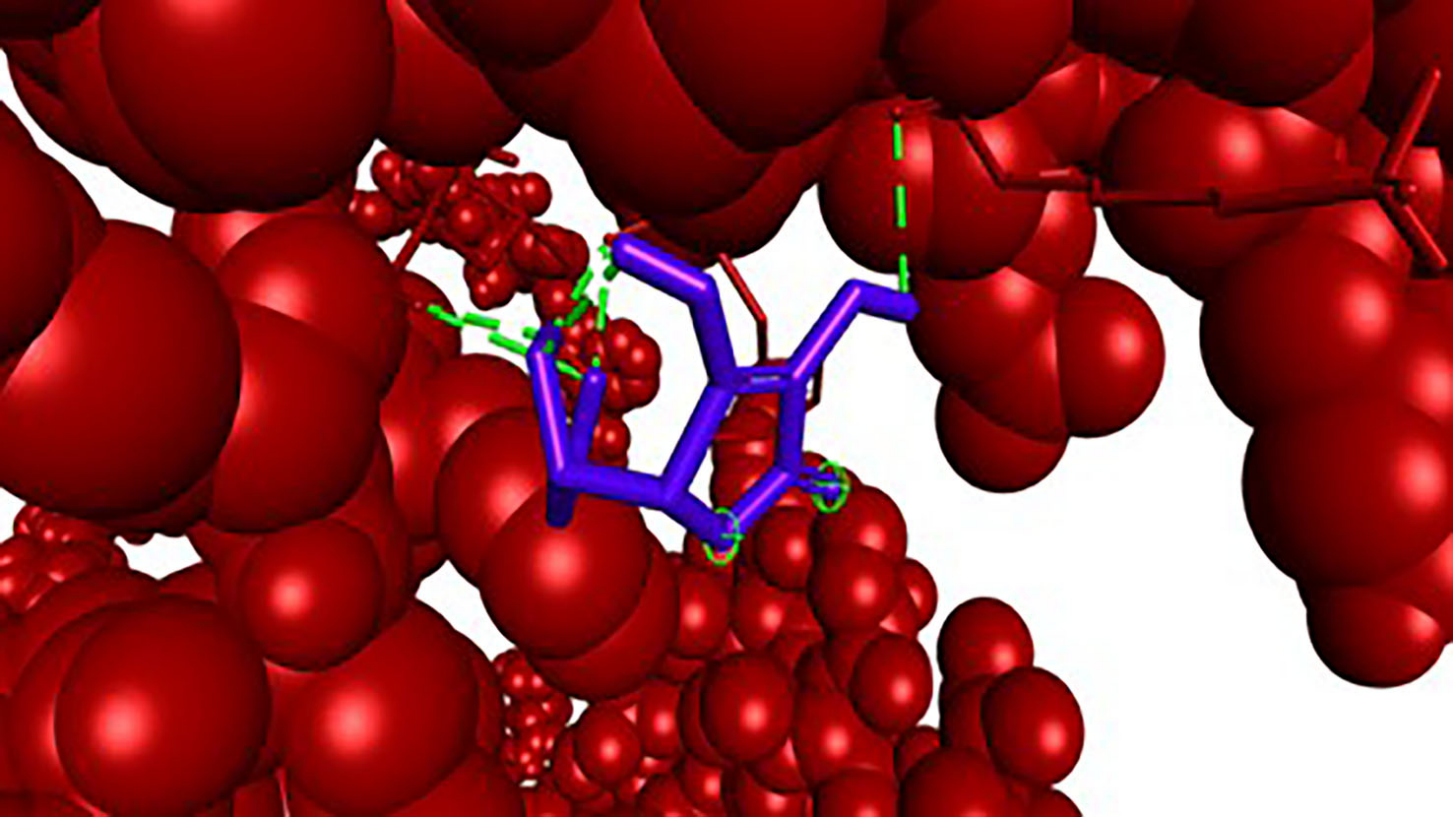
Closer view interaction between NRF2 protein and ligand (Vitamin C).

All six compounds were subjected to molecular docking against Vitamin C and testosterone to determine which exhibited the strongest binding affinity. Using the AutoDock Vina algorithm within the PyRx platform, 9,12,15-Octadecatrienoic acid emerged as the compound with the highest binding affinity. Consequently, ligands of 9,12,15-Octadecatrienoic acid, Vitamin C, and testosterone were selected for further protein–ligand interaction analysis. Binding interactions with the target proteins—Nuclear factor erythroid 2–related factor 2 (NRF2) and the Androgen Receptor (AR)—were evaluated using Discovery Studio Visualizer. The analysis highlighted key interactions such as hydrogen bonding, van der Waals forces, and hydrophobic contacts that contribute to the stability and specificity of ligand–protein binding ( Figures 2- 4).

**Figure 5.**
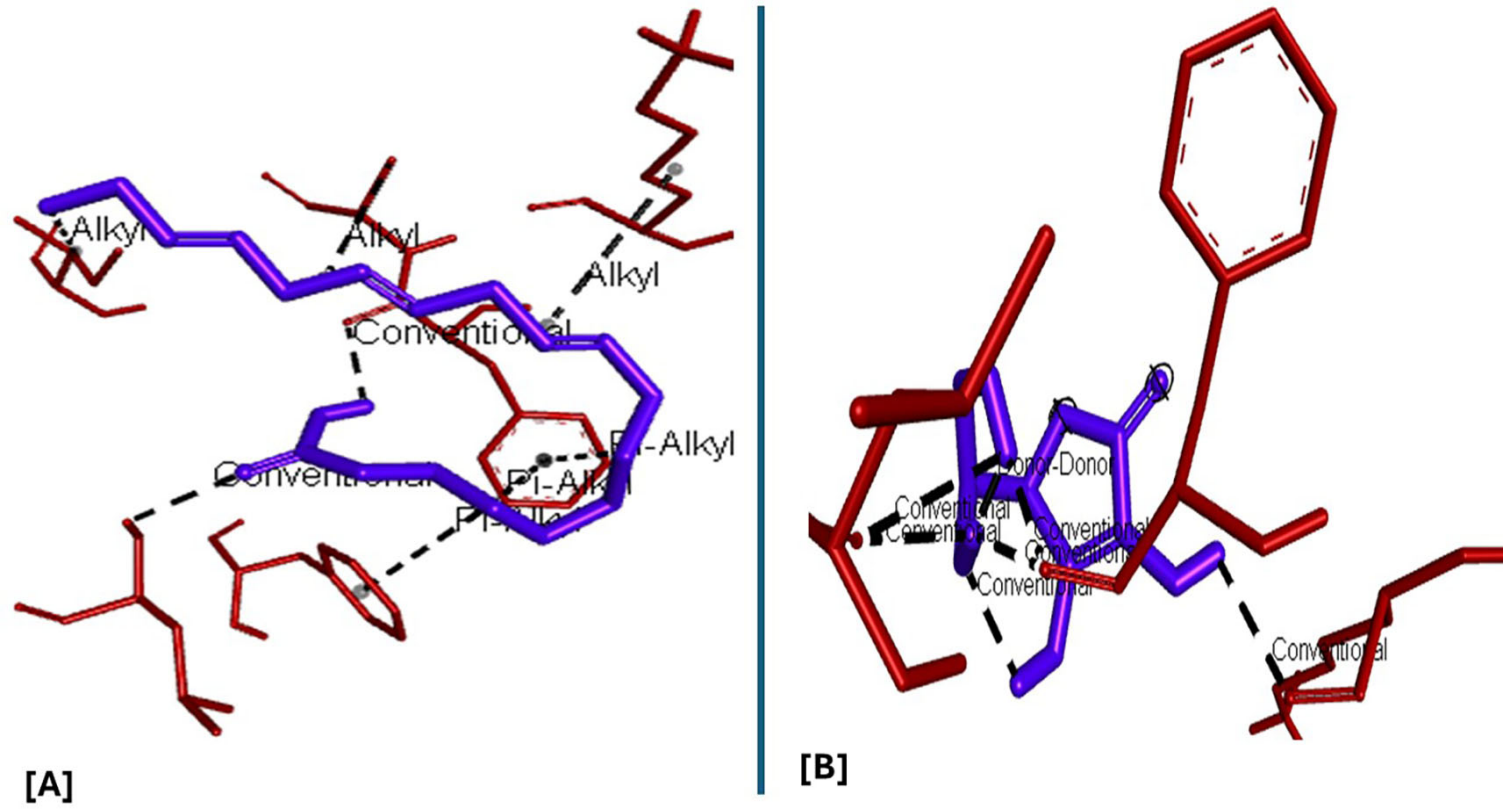
3D interaction showing the types of bond between NRF2 protein and ligand. A = ligand (9,12,15-Octadecatrienoic acid), B = ligand (Vitamin C).

**Figure 6.**
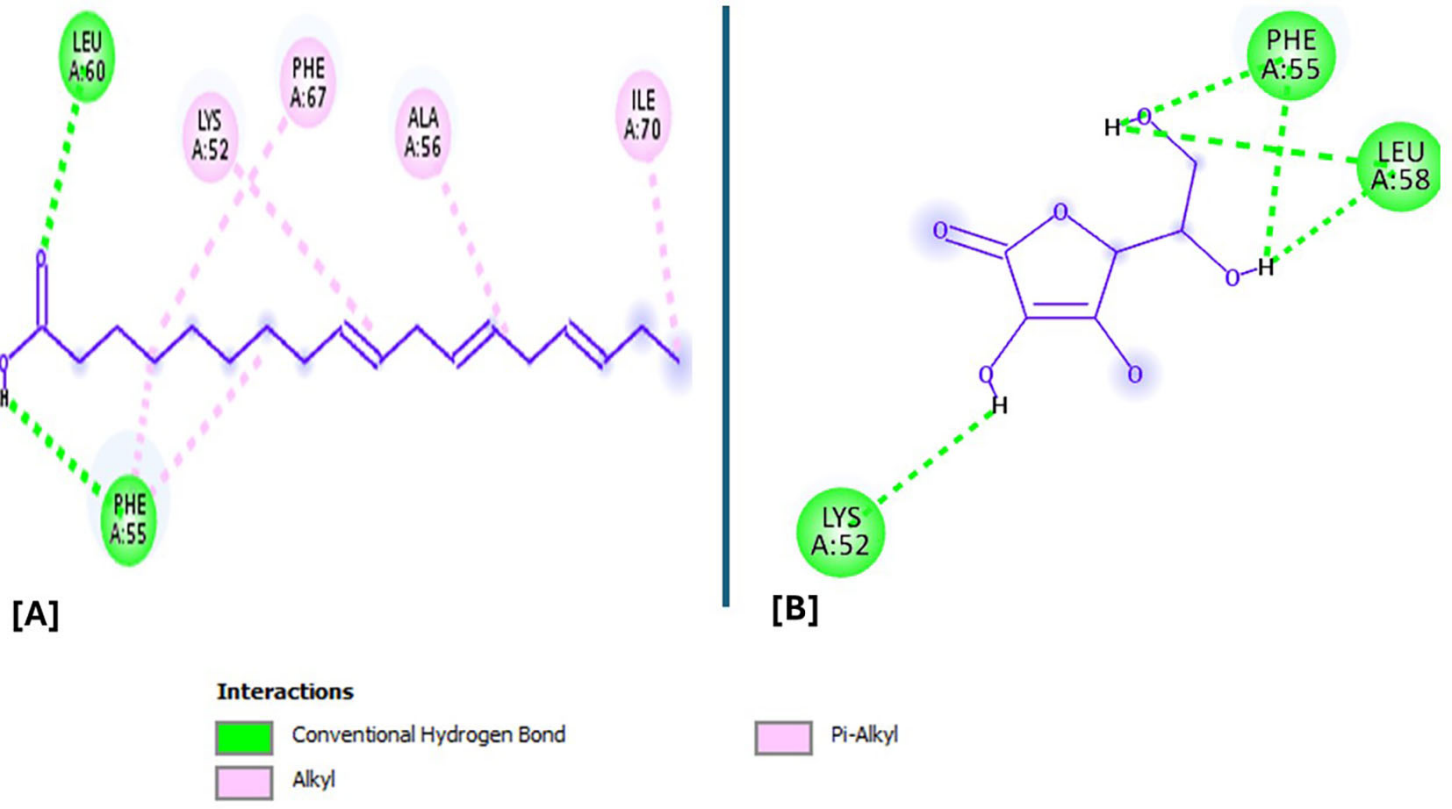
2D interaction illustrating the types of bonds. The compounds form conventional hydrogen bonds with the protein at Leucine and Phenyl. A= ligand (9,12,15-Octadecatrienoic acid), B= ligand (Vitamin C).

Analysis of the three-dimensional protein–ligand complexes revealed that both 9,12,15-octadecatrienoic acid and vitamin C bind to the same active site on NRF2. Two-dimensional interaction diagrams confirmed the presence of conventional hydrogen bonds between the ligands and NRF2. Specifically, 9,12,15-octadecatrienoic acid formed hydrogen bonds with Phe55 and Leu60, while vitamin C interacted with Phe55, Leu58, and Lys52 ( Figures 5- 6). Given that vitamin C is a well-established antioxidant, the similar binding pattern observed with 9,12,15-octadecatrienoic acid suggests that it may also exhibit antioxidant activity.

**Figure 7.**
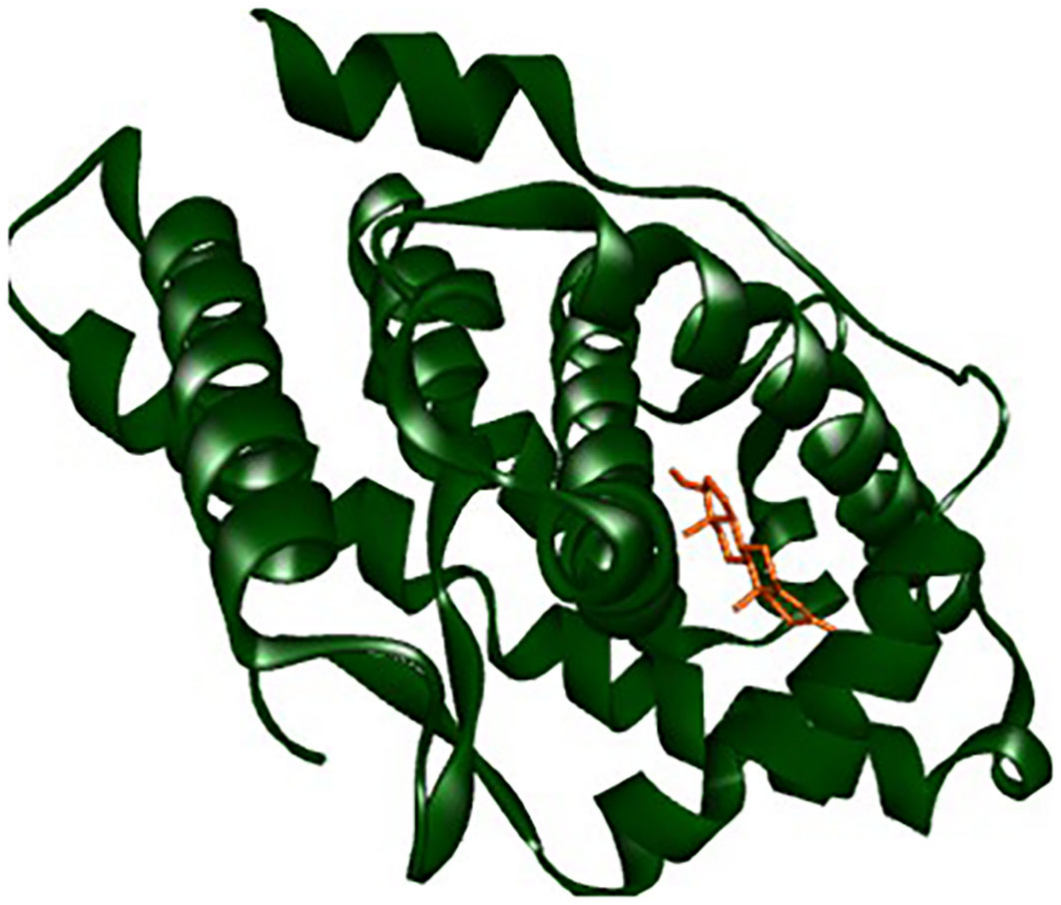
AR protein and its binding site.

**Figure 8.**
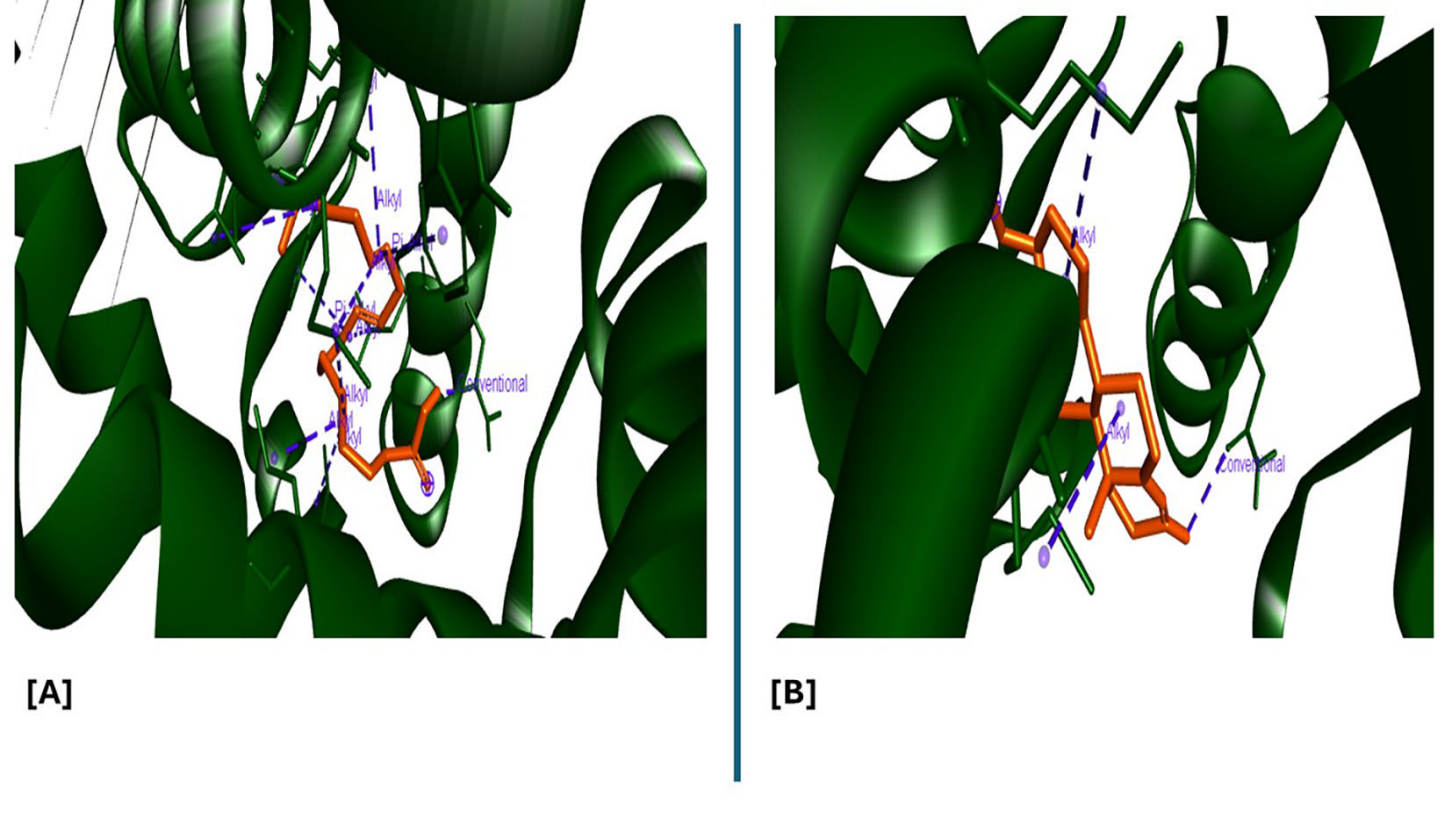
Closer view interaction between AR protein and ligands. A = ligand (9,12,15-Octadecatrienoic acid), B = ligand (Testosterone).

**Figure 9.**
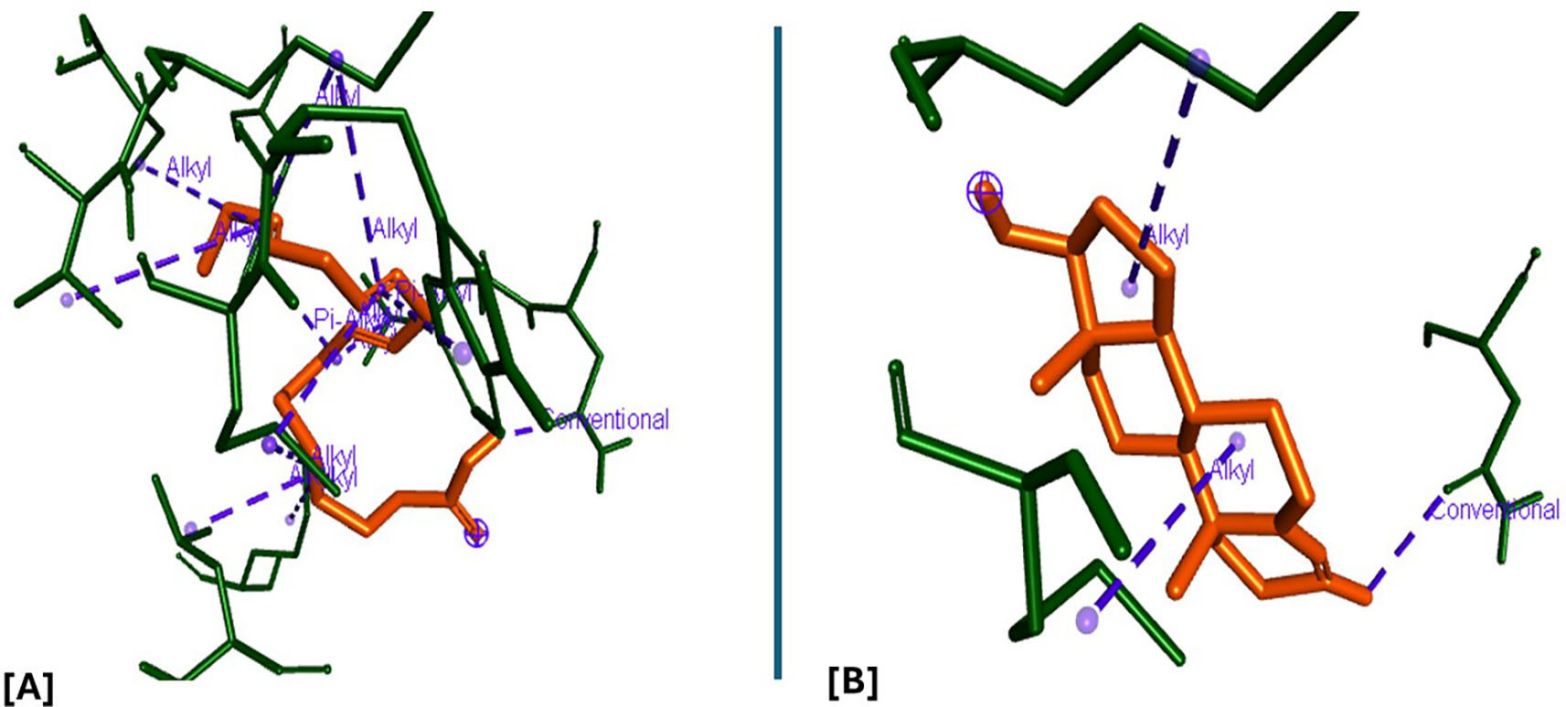
3D interaction showing the types of bond between AR protein and ligands. A= ligand (9,12,15-Octadecatrienoic acid), B= ligand (Testosterone).

**Figure 10.**
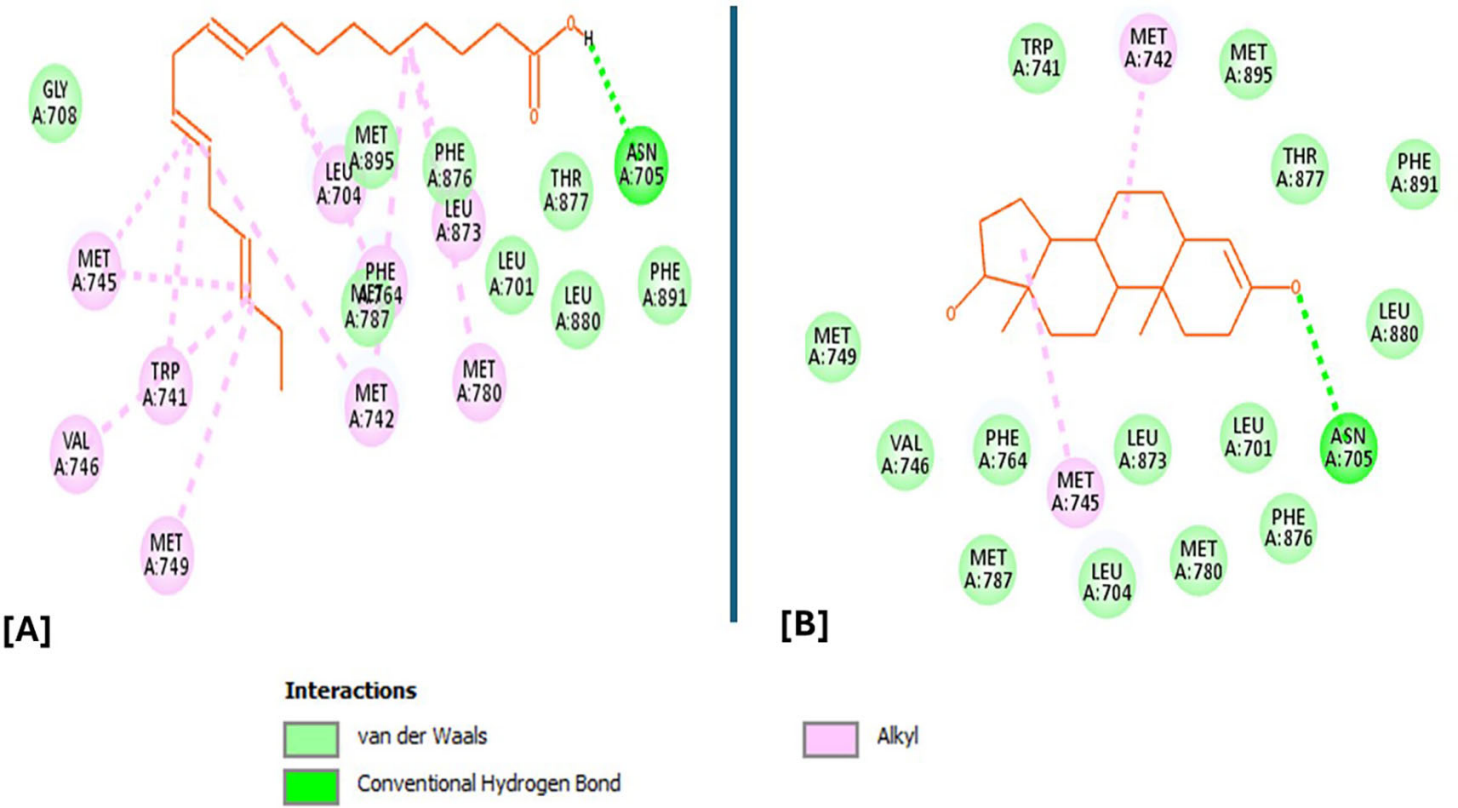
2D interaction illustrating the types of bonds. The compounds form conventional hydrogen bonds with the protein at several binding sites. A = ligand (9,12,15-Octadecatrienoic acid), B = ligand (Testosterone).

Analysis of the three-dimensional protein–ligand complexes showed that both 9,12,15-octadecatrienoic acid and testosterone bind to the same active site on the androgen receptor (AR) ( Figures 7- 8). Two-dimensional interaction diagrams revealed the presence of conventional hydrogen bonds and alkyl interactions between the ligands and AR ( Figures 9- 10). Since testosterone and related androgens exert their physiological effects by binding to AR and regulating genes critical for spermatogenesis, sperm maturation, and overall testicular function, the observed binding pattern suggests that 9,12,15-octadecatrienoic acid may mimic androgenic activity and influence reproductive function.

### DPPH radical scavenging

**Figure 11.**
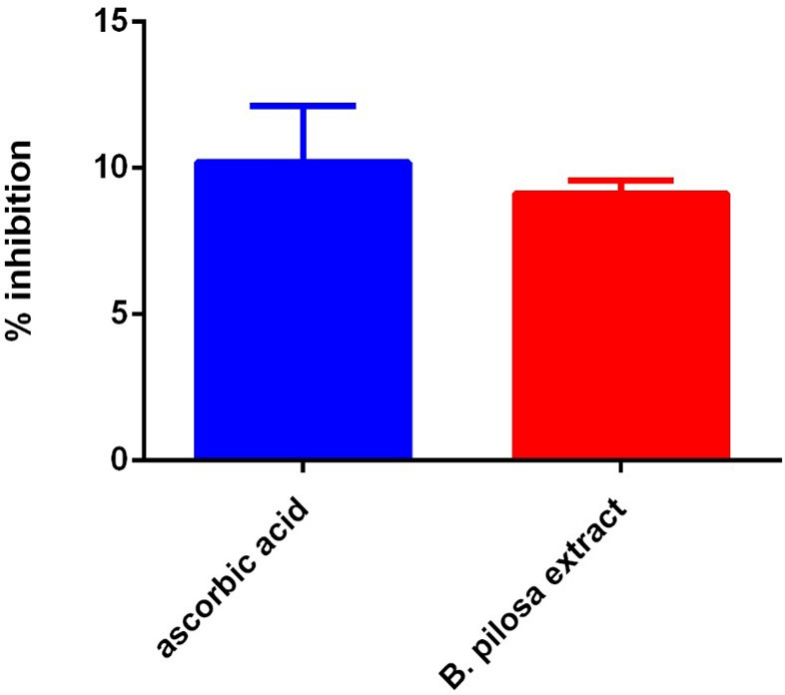
DPPH radical scavenging activities of *B. pilosa* and Vitamin C. Values are average of duplicate experiment and represented as mean ± SEM.

This analysis compares the antioxidant activity of the ethyl acetate fraction of *B. pilosa* to that of ascorbic acid (vitamin C) using the DPPH free radical scavenging assay, where a lower IC _50_ value indicates stronger antioxidant activity. The DPPH radical is decolorized upon reduction, and this change can be quantitatively measured by the decrease in absorbance at 515–517 nm. The IC _50_ values of vitamin C and the ethyl acetate extract of *B. pilosa* are presented in Figure 11. Vitamin C exhibited the highest scavenging activity with an IC _50_ of 1.25 μg/ml, indicating strong antioxidant potential. In comparison, the *B. pilosa* extract showed moderate antioxidant activity, with an IC _50_ of 6.11 μg/ml. Although less potent than vitamin C, the extract still demonstrated notable free radical scavenging ability ( Figure 11).

### Weight change

**Figure 12.**
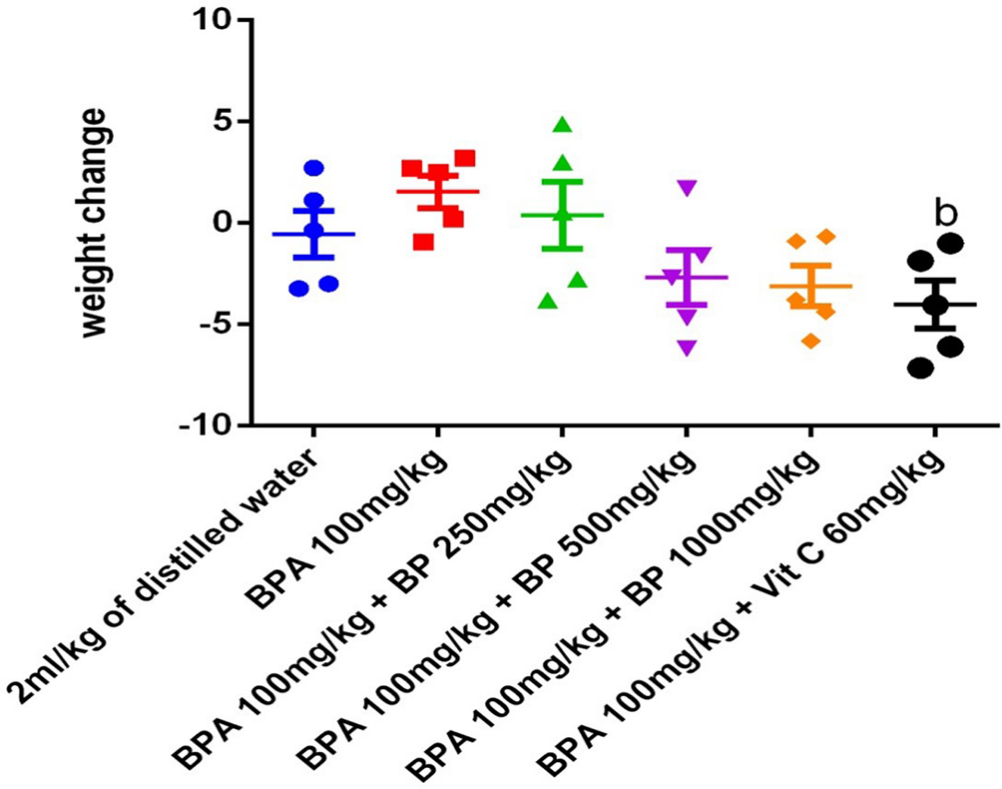
Effect of *Bidens pilosa* extract-treatment and drug treatment on body weight (g/mice) of mice exposed to Bisphenol A. Data are expressed as mean ± SEM, n = 5. **b** indicates significant difference from the group administered BPA at 100 mg/kg/day (p < 0.05).

Following five weeks of treatment, body weight was reduced in the control group (2 mL/kg distilled water), as well as in the groups treated with 250 mg/kg (Group 3), 500 mg/kg (Group 4), and 1000 mg/kg (Group 5) of the ethyl-acetate extract of *Bidens pilosa*, and 60 mg/kg of vitamin C (Group 6), when compared to the BPA-only group (100 mg/kg/day, Group 2). Notably, the group treated with 60 mg/kg of vitamin C (Group 6) exhibited a statistically significant reduction in body weight (p ≤ 0.05) compared to the BPA-treated group, suggesting a possible modulation of BPA-induced metabolic effects ( Figure 12).

### Antioxidant enzyme level

**Figure 13.**
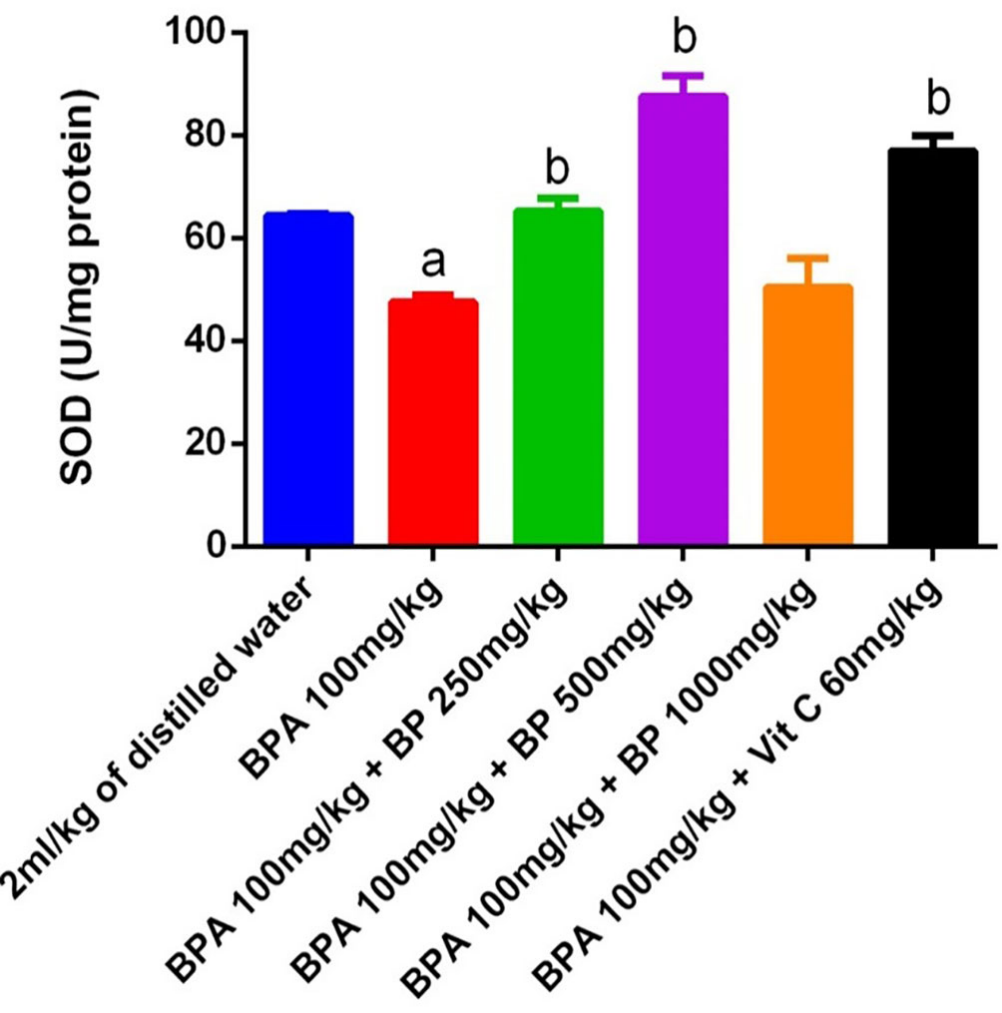
SOD activities in BPA-induced mice treated with *Bidens pilosa* leaf extract and Vitamin C. **a** denotes a significant difference from the control group; **b** denotes a significant difference from the Bisphenol A group (p < 0.05). BPA – Bisphenol A (n = 3).

Superoxide dismutase (SOD) activity in the testes and epididymis of mice is shown in Figure 13. A significant reduction (p < 0.05) in SOD activity was observed in the BPA-induced group compared to the control, indicating oxidative stress. However, treatment with *Bidens pilosa* extract at doses of 250 mg/kg and 500 mg/kg, as well as with 60 mg/kg of vitamin C, significantly restored SOD activity relative to the BPA group (p < 0.05). While extract- and vitamin C-treated groups exhibited slightly higher SOD activity than the control, these increases were not statistically significant.

**Figure 14.**
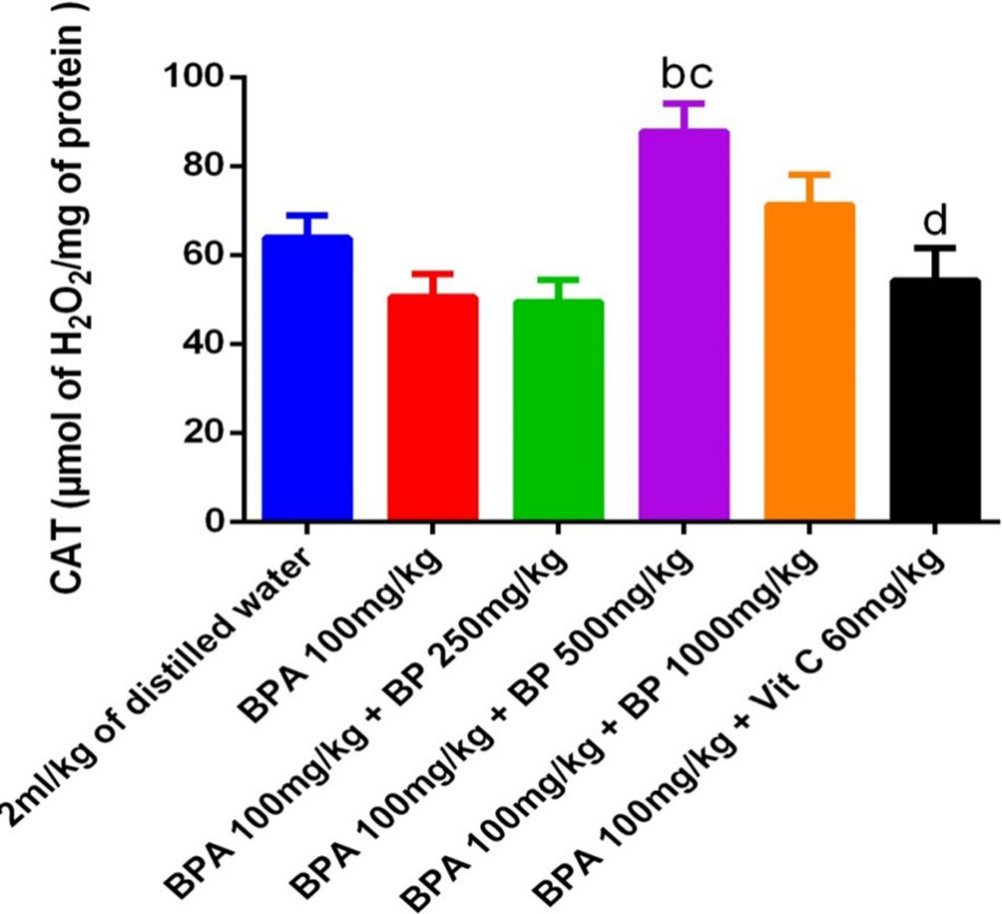
CAT activities in BPA-induced mice treated with *Bidens pilosa* leaf extract and Vitamin C. **a** denotes a significant difference from the control group; **b** denotes a significant difference from the BPA 100 mg/kg group; **c** denotes a significant difference from the group treated with BPA 100 mg/kg +250 mg/kg leaf extract; **d** denotes a significant difference from the group treated with BPA 100 mg/kg +500 mg/kg leaf extract (p < 0.05).

Figure 14 presents the catalase (CAT) activity in the testes. Although there was no statistically significant difference in CAT activity between the BPA-induced and control groups, a modest reduction was noted in the BPA group. Treatment with 500 mg/kg of *B. pilosa* extract resulted in a significant increase in CAT activity compared to the BPA group (p < 0.05), though this improvement did not reach significance relative to the control.

### Oxidative stress biomarker

**Figure 15.**
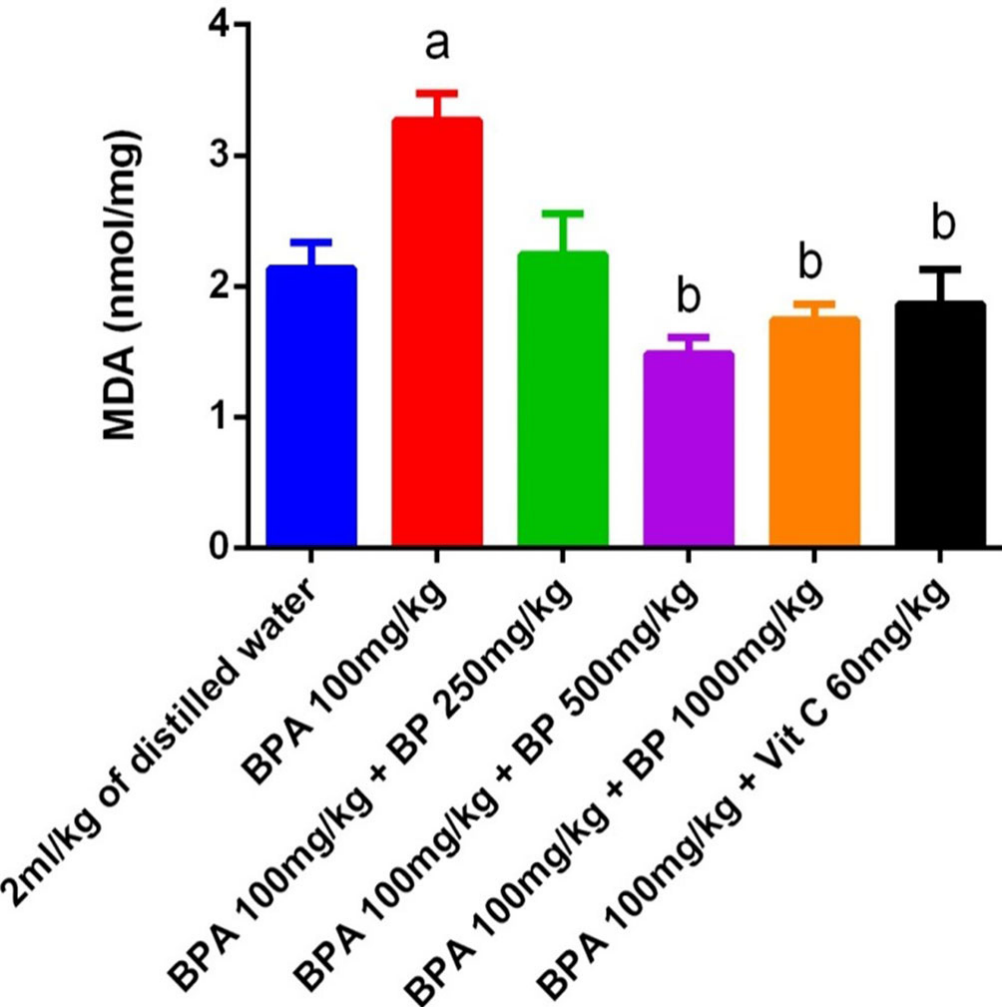
MDA activities in BPA-induced mice treated with *Bidens pilosa* leaf extract and Vitamin C. **a** denotes a significant difference from the control group; **b** denotes a significant difference from the Bisphenol A group (p < 0.05). BPA – Bisphenol A (n = 3).

Malondialdehyde (MDA) levels, a marker of lipid peroxidation, are displayed in Figure 15. MDA levels were significantly elevated (p < 0.05) in the BPA group relative to the control, confirming oxidative damage. Administration of *B. pilosa* extract at 500 mg/kg and 1000 mg/kg, as well as vitamin C, led to a significant reduction in MDA levels compared to the BPA-induced group (p < 0.05), indicating protective antioxidant effects.

## Discussion

The present study investigated the antioxidant and reproductive protective potential of the ethyl acetate fraction of *Bidens pilosa* leaves in male mice exposed to bisphenol A (BPA), a known endocrine disruptor. The research combined biochemical assays, free radical scavenging studies, and molecular docking to elucidate the mechanisms by which *B. pilosa* and its phytoconstituents might mitigate oxidative and reproductive damage.

Oxidative stress plays a central role in the pathophysiology of BPA-induced reproductive toxicity, as it disrupts the delicate redox balance essential for normal testicular function. ^23^ In the present study, BPA exposure significantly impaired testicular antioxidant defenses, evidenced by decreased activity of superoxide dismutase (SOD) and elevated levels of malondialdehyde (MDA), a key end product of lipid peroxidation. These alterations indicate an accumulation of reactive oxygen species (ROS) and peroxidative damage to membrane lipids, ultimately compromising the structural and functional integrity of germ cells, which is consistent with earlier reports that implicate oxidative stress as a major driver of BPA-mediated testicular injury and infertility. ^24^ Treatment with the ethyl acetate fraction of *Bidens pilosa* effectively countered these disturbances in a dose-dependent manner, since both 250 mg/kg and 500 mg/kg enhanced SOD activity, with the higher dose producing a more pronounced effect. Restoration of SOD activity is particularly important because this enzyme constitutes the first line of enzymatic defense against oxidative damage by catalyzing the dismutation of superoxide anions into hydrogen peroxide, and by reactivating this pathway, *B. pilosa* extract likely prevented the amplification of oxidative cascades that exacerbate testicular cell injury. Similarly, administration of *B. pilosa* significantly reduced MDA levels, which indicates decreased lipid peroxidation and preservation of cellular membranes, reflecting the extract’s ability to neutralize ROS before they attack polyunsaturated fatty acids in cell membranes, a process known to trigger germ cell apoptosis and impair spermatogenesis. This protective effect may be attributed to the synergistic action of *B. pilosa* phytochemicals, such as flavonoids and fatty acid derivatives, which are known to quench free radicals and stabilize redox-sensitive cellular pathways. ^25, 26^ Interestingly, catalase (CAT) activity showed a different pattern because BPA exposure did not significantly reduce CAT levels compared to controls, yet treatment with *B. pilosa*, particularly at 500 mg/kg, significantly upregulated CAT activity. This suggests that the extract not only restores enzymes inhibited by BPA but also strengthens antioxidant defenses that were not directly suppressed, and because CAT is responsible for decomposing hydrogen peroxide into water and oxygen, its upregulation implies that *B. pilosa* may enhance the downstream detoxification of ROS, complementing the effects of SOD. This dual enhancement of SOD and CAT highlights the extract’s capacity to fortify the endogenous antioxidant network, providing a more comprehensive defense against oxidative insults. Taken together, these findings suggest that the ethyl acetate extract of *B. pilosa* mitigates BPA-induced oxidative stress through the reactivation of suppressed antioxidant enzymes such as SOD, attenuation of lipid peroxidation as reflected in reduced MDA levels, and upregulation of non-suppressed antioxidant enzymes such as CAT, thereby strengthening the overall antioxidant response and preventing secondary oxidative cascades. These antioxidant effects are crucial because persistent oxidative stress in the testes can trigger apoptosis of germ cells, disruption of the blood-testis barrier, and impaired steroidogenesis, all of which contribute to reduced fertility, and by stabilizing redox homeostasis, *B. pilosa* extract may therefore protect not only the structural integrity of the testes but also their functional capacity to support spermatogenesis and hormonal balance.

Beyond its modulatory effects on oxidative stress, BPA exposure also produced notable alterations in body weight, as BPA-treated mice exhibited significant weight gain compared to the control group. This observation is consistent with accumulating evidence that BPA functions as an “obesogen,” disrupting normal energy homeostasis and promoting lipid accumulation through mechanisms involving endocrine interference, mitochondrial dysfunction, and altered adipocyte differentiation. ^27, 28^ In particular, BPA has been shown to interact with estrogen receptors, peroxisome proliferator-activated receptors (PPARs), and other nuclear receptors that regulate lipid metabolism and glucose homeostasis, thereby predisposing exposed animals to increased adiposity and metabolic dysfunction.

In contrast, administration of the *B. pilosa* extract, as well as vitamin C, reversed this abnormal weight gain. While the vitamin C group showed a statistically significant reduction in body weight (p ≤ 0.05), the extract-treated groups also demonstrated a clear downward trend, suggesting protective or corrective effects on BPA-induced metabolic dysregulation. This effect may be attributed to the presence of bioactive phytochemicals in *B. pilosa*, such as flavonoids, phytosterols, and fatty acid derivatives, which have been reported to modulate lipid metabolism, enhance insulin sensitivity, and attenuate low-grade systemic inflammation. ^29^ By influencing pathways that regulate adipogenesis and lipid oxidation, these compounds could mitigate the metabolic imbalance triggered by BPA exposure.

Furthermore, phytochemicals such as stigmasterol and phytol identified in the extract may exert hypolipidemic effects by modulating hepatic cholesterol metabolism and suppressing inflammatory cytokines that contribute to obesity-associated metabolic syndrome. ^23^ The ability of *B. pilosa* to prevent excessive weight gain may therefore reflect a combined action of antioxidant protection, anti-inflammatory signaling, and regulation of metabolic pathways linked to energy utilization and fat storage.

The bioactive constituents identified in the ethyl acetate fraction of *B. pilosa* may exert their protective actions through interactions with specific molecular targets that regulate oxidative stress, inflammation, and reproductive signaling. For instance, 9,12,15-octadecatrienoic acid (alpha-linolenic acid) has been shown to modulate nuclear factor erythroid 2–related factor 2 (Nrf2) signaling, thereby promoting the transcription of downstream antioxidant response genes such as heme oxygenase-1 and glutathione peroxidase. ^30^ By enhancing Nrf2 activation, this compound reduces ROS accumulation and preserves cellular redox homeostasis. ^31^ In addition, docking studies in this work revealed strong interactions between 9,12,15-octadecatrienoic acid and the androgen receptor (AR), suggesting a direct role in supporting androgenic signaling critical for spermatogenesis.

Collectively, phytochemicals in *B. pilosa* act on interconnected molecular targets ( **Nrf2** activation) to enhance antioxidant capacity, reduce inflammatory stress, regulate metabolic balance, and AR modulation preserves reproductive signaling. Such a multi-target mode of action highlights the synergistic interplay of *B. pilosa* phytoconstituents, explaining the broad-spectrum ameliorative effects observed in BPA-exposed mice. This integrative mechanism reinforces the therapeutic potential of *B. pilosa* as a natural intervention capable of addressing oxidative, metabolic, and reproductive disturbances simultaneously.

The integration of biochemical, in vitro, and in silico findings highlights a dual mechanism of action for *Bidens pilosa*, involving direct antioxidant activity through the enhancement of enzymatic defenses and attenuation of lipid peroxidation, alongside hormonal and signaling modulation via phytoconstituent interactions with the androgen receptor and other redox-sensitive pathways, which together contribute to preserving testicular integrity and reproductive potential in the face of BPA-induced stress. ^32^

While the findings are promising, several limitations must be acknowledged. The antioxidant profile was limited to DPPH, excluding assays such as ABTS, FRAP, and ORAC, which would have provided a broader perspective. Molecular docking results were not validated by molecular dynamics simulations due to computational resource limitations. Moreover, downstream analyses of NRF2 and AR signaling, as well as functional reproductive outcomes such as sperm quality and fertility, were not assessed. Future studies should incorporate these analyses to establish a more direct link between molecular mechanisms and reproductive function.

## Conclusion

This study demonstrates that the ethyl-acetate extract of *Bidens pilosa* provides significant protection against BPA-induced oxidative stress and reproductive toxicity, with evidence supporting both antioxidant enhancement and androgenic modulation as underlying mechanisms. By integrating biochemical assays, free radical scavenging studies, and molecular docking, the work establishes a mechanistic basis for the extract’s protective actions, identifying specific phytoconstituents such as 9,12,15-octadecatrienoic acid, phytol, hexadecanoic acid, and stigmasterol as potential contributors. These findings not only expand current understanding of BPA-induced reproductive dysfunction but also position *B. pilosa* as a promising natural candidate for mitigating oxidative and endocrine-disrupting effects of environmental toxicants. Although further research is warranted including additional antioxidant assays, molecular dynamics simulations, gene expression analyses, and functional reproductive assessments, this study provides an important foundation for the development of phytotherapeutic interventions against male reproductive impairment.

## Data Availability

Figshare. Male Fertility enhancing potential of Biden pilosa during Bisphenol A exposure. https://doi.org/10.6084/m9.figshare.20893240.v2. ^14^ This project contains the following underlying data:
• DPPH Result. (Contain the DPPH result) • DATA_B. pilosaPhD. (Sheet 3 Contain the result of the oxidative stress study result) • DATA_B. pilosaPhD. (Sheet 1 Contain the result of the body weight change) Data are available under the terms of the Creative Commons Attribution 4.0 International license (CC-BY 4.0). Figshare: Arrive checklist for “Male Fertility enhancing potential of Biden pilosa during Bisphenol A exposure”. DOI: https://doi.org/10.6084/m9.figshare.20893240.v2. ^14^ Data are available under the terms of the Creative Commons Attribution 4.0 International license (CC-BY 4.0).

## References

[1] Eisenberg ML : Options after a failed microsurgical testicular sperm extraction. *Fertil. Steril.* 2023;120(2):240–241. 10.1016/j.fertnstert.2023.05.166 37268045

[2] Dutta S Sengupta P Slama P : Oxidative stress, testicular inflammatory pathways, and male reproduction. *Int. J. Mol. Sci.* 2021;22(18):10043. 10.3390/ijms221810043 34576205 PMC8471715

[3] Ahmad I Kaur M Tyagi D : Exploring novel insights into the molecular mechanisms underlying bisphenol a-induced toxicity: A persistent threat to human health. *Environ. Toxicol. Pharmacol.* 2024;108:104467. 10.1016/j.etap.2024.104467 38763439

[4] Pan J Liu P Yu X : The adverse role of endocrine disrupting chemicals in the reproductive system. *Front. Endocrinol. (Lausanne).* 2024;14:1324993. 10.3389/fendo.2023.1324993 38303976 PMC10832042

[5] Bey A ArrvinD A Yadav PK : Malondialdehyde: A Toxic Stress Marker for Periodontitis. *J. Clin. Diagn. Res.* 2024;18(3). 10.7860/JCDR/2024/68440.19195

[6] Saxena P Selvaraj K Khare SK : Superoxide dismutase as multipotent therapeutic antioxidant enzyme: Role in human diseases. *Biotechnol. Lett.* 2022;44:1–22. 10.1007/s10529-021-03200-3 34734354

[7] Sharafi K Kiani A Massahi T : Bisphenol a (BPA) emitted from food cans: an evaluation of the effects of dry heating, boiling, storage period, and food type on migration and its potential impact on human health. *Int. J. Environ. Anal. Chem.* 2024;104(20):9246–9259. 10.1080/03067319.2023.2228701

[8] Shawon SI Reyda RN Qais N : Medicinal herbs and their metabolites with biological potential to protect and combat liver toxicity and its disorders: A review. *Heliyon.* 2024;10(3):e25340. 10.1016/j.heliyon.2024.e25340 38356556 PMC10864916

[9] Adeniyi IA Olufunke O Usman IM : UNRAVELING THE REPRODUCTIVE POTENTIAL OF SELECTED FLAVONOIDS IN BIDEN PILOSA: A COMPREHENSIVE REVIEW. *Phytomed. Plus.* 2025;5:100784. 10.1016/j.phyplu.2025.100784

[10] Bandonienė D Murkovic M Pfannhauser W : Detection and activity evaluation of radical scavenging compounds by using DPPH free radical and on-line HPLC-DPPH methods. *Eur. Food Res. Technol.* 2002;214:143–147. 10.1007/s00217-001-0430-9

[11] Morris GM : Docking and Virtual Screening. 2012.

[12] Santello M Toni N Volterra A : Astrocyte function from information processing to cognition and cognitive impairment. *Nat. Neurosci.* 2019;22(2):154–166. 10.1038/s41593-018-0325-8 30664773

[13] Usman IM Adebisi SS Musa SA : Neurobehavioral and Immunohistochemical Studies of the Cerebral Cortex Following Treatment with Ethyl Acetate Leaf Fraction of Tamarindus indica During Prenatal Aluminum Chloride Exposure in Wistar Rats. *J. Exp. Pharmacol.* 2022;14:275–289. 10.2147/JEP.S369631 36303592 PMC9592736

[14] Usman MI Adeniyi IA : Male Fertility enhancing potential of Biden pilosa during Bisphenol A exposure. *Figshare.* 10.6084/m9.figshare.20893240.v2

[15] Alekhya Sita GJ Gowthami M Srikanth G : Protective role of luteolin against bisphenol A-induced renal toxicity through suppressing oxidative stress, inflammation, and upregulating Nrf2/ARE/HO-1 pathway. *IUBMB Life.* 2019;71(7):1041–1047. 10.1002/iub.2066 31091348

[16] Lagat JK Ng’wena AGM Mwaniki DM : Effects of Achyranthes aspera, Bidens pilosa and Ajuga remota leaf extracts on serum glucose and electrolyte levels in alloxan treated male goats. *Afr. J. Health Sci.* 2021;34(4):537–549.

[17] Jimoh AG Folorunsho AS Olatunbosun A : Evaluating the Supplementary Effects of Vitamin C on Carbamazepine and Pentylenetetrazol-Induced Seizures and Preimplantation Loss in Pregnant Wistar Rats: Implications for Human Pregnancy. *Asia Pacific J. Med. Toxicol.* 2024;13(2).

[18] Théophile D Emery TD Desire DDP : Effects of Alafia multiflora Stapf on lipid peroxidation and antioxidant enzyme status in carbon tetrachloride treated rats. *Pharmacol. Online.* 2006;2:76–89.

[19] Kiran U Kumar K Roy A : An intelligent dimension-based cat swarm optimization for efficient cooperative multi-hop relay selection in vehicular network. *Neural Comput. & Applic.* 2023;35(21):15381–15395. 10.1007/s00521-023-08541-w

[20] Kilford PJ Chen K Crewe K : Prediction of CYP-mediated DDIs involving inhibition: approaches to address the requirements for system qualification of the Simcyp simulator. *CPT Pharmacometrics Syst. Pharmacol.* 2022;11(7):822–832. 10.1002/psp4.12794 35445542 PMC9286715

[21] Wollmann BM Størset E Kringen MK : Prediction of CYP2D6 poor metabolizers by measurements of solanidine and metabolites—a study in 839 patients with known CYP2D6 genotype. *Eur. J. Clin. Pharmacol.* 2023;79(4):523–531. 10.1007/s00228-023-03462-y 36806969 PMC10038974

[22] Park JN Tardif J Thompson E : A survey of North American drug checking services operating in 2022. *Int. J. Drug Policy.* 2023;121:104206. 10.1016/j.drugpo.2023.104206 37797571 PMC10843152

[23] Meli R Monnolo A Annunziata C : Oxidative stress and BPA toxicity: an antioxidant approach for male and female reproductive dysfunction. *Antioxidants.* 2020;9(5):405. 10.3390/antiox9050405 32397641 PMC7278868

[24] Wang Y Fu X Li H : Mechanisms of oxidative stress-induced sperm dysfunction. *Front. Endocrinol (Lausanne).* 2025;16:1520835. 10.3389/fendo.2025.1520835 39974821 PMC11835670

[25] Akinmoladun AC Ibukun EO Afor E : Phytochemical constituent and antioxidant activity of extract from the leaves of Ocimum gratissimum. *Sci. Res. Essay.* 2007;2(5):163–166.

[26] Omoregie ES Oriakhi K Oikeh EI : Comparative study of phenolic content and antioxidant activity of leaf extracts of Alstonia boonei and Eupatorium odoratum. *Niger. J. Basic Appl. Sci.* 2014;22(3-4):91–97.

[27] Rochester JR : Bisphenol A and human health: A review of the literature. *Reprod. Toxicol.* 2013;42:132–155. 10.1016/J.REPROTOX.2013.08.008 23994667

[28] Dolatabadi S Oskuei SR Mehri S : A comprehensive review of medicinal plants and their beneficial roles in alleviating bisphenol A–induced organ toxicity. *Naunyn Schmiedebergs Arch. Pharmacol.* 2025;398:7801–7876. 10.1007/s00210-025-03795-8 39932506

[29] Everds NE Snyder PW Bailey KL : Interpreting stress responses during routine toxicity studies: a review of the biology, impact, and assessment. *Toxicol. Pathol.* 2013;41(4):560–614. 10.1177/0192623312466452 23475558

[30] Bejarano E Weinberg J Clark M : Redox regulation in age-related cataracts: roles for glutathione, vitamin C, and the NRF2 signaling pathway. *Nutrients.* 2023;15(15):3375. 10.3390/nu15153375 37571310 PMC10421530

[31] Rotimi DE Ojo OA Olaolu TD : Exploring Nrf2 as a therapeutic target in testicular dysfunction. *Cell Tissue Res.* 2022;390(1):23–33. 10.1007/s00441-022-03664-3 35788899

[32] Dyall SC Balas L Bazan NG : Polyunsaturated fatty acids and fatty acid-derived lipid mediators: Recent advances in the understanding of their biosynthesis, structures, and functions. *Prog. Lipid Res.* 2022;86:101165. 10.1016/j.plipres.2022.101165 35508275 PMC9346631

