# Supplementary material for: Ameliorative Effects of Ethyl-Acetate Extract of Bidens pilosa on Oxidative Stress and Reproductive Impairment in Bisphenol A-Exposed Male Mice: Insight from In-silico, Invitro and In-vivo Studies
Source: F1000Res. 2025 Dec 2;14:771. Originally published 2025 Aug 6. [Version 2] doi: 10.12688/f1000research.166336.2 (PMC12811727; doi:10.12688/f1000research.166336.2)
Supplement: Peer review history

# Reviewer report: Reviewer response for version 2

**DOI:** 10.5256/f1000research.192058.r449875

**Published:** 2026-01-16

**Authors:** Mustofa Ahda

**Affiliations:** 1 Universitas Ahmad Dahlan, Yogyakarta, Special Region of Yogyakarta, Indonesia

Dear Authors

 I recommended that this paper be revised totally. You need to revise some parts as mentioned below:

 1) Profiling of chemical compounds of Ethyl-Acetate Extract of *Bidens pilosa and its *Ameliorative Effects on Oxidative Stress and Reproductive Impairment in Bisphenol A-Exposed Male Mice based on In silico approach and In vivo evaluation

 2) please revise your abstract. you must elaborate the potency of Bident Pilosa or the increase of the oxidantive stress must be treated

 3) please add gap analysis in the introduction

 4) why do you only evaluate the volatile compounds. I recommended for you analyse using HRMS

 5) why do you only analyse DPPH inhibition. I recommended to analyse ABTS inhibition or FRAP

 6) Why did not you mixe the Table 2 and 3 become 1 Table

 7) please create 1 Figure 2 and 7 and Figure 3, 4 and Figure 8,9

 Please create 1 figure between figure 5, 6 and Figure 9,10

 8) please create 1 figure between figure 13,14, 15

Is the work clearly and accurately presented and does it cite the current literature?

No

If applicable, is the statistical analysis and its interpretation appropriate?

Yes

Are all the source data underlying the results available to ensure full reproducibility?

Yes

Is the study design appropriate and is the work technically sound?

No

Are the conclusions drawn adequately supported by the results?

No

Are sufficient details of methods and analysis provided to allow replication by others?

No

Reviewer Expertise:

Herbal analysis

I confirm that I have read this submission and believe that I have an appropriate level of expertise to confirm that it is of an acceptable scientific standard, however I have significant reservations, as outlined above.

# Reviewer report: Reviewer response for version 1

**DOI:** 10.5256/f1000research.183308.r410192

**Published:** 2025-09-24

**Authors:** Parween Abdulsamad Ismail

**Affiliations:** 1 University of Salahaddin, Erbil, Iraq

*This is a well-written manuscript that only needs to undergo a few minor changes. :*

 Only minor revisions are needed before it can be indexed.

 *This discussion could be expanded to explain the Ameliorative Effects of Ethyl-Acetate Extract of *Bidens pilosa* on Oxidative Stress and Reproductive Impairment

  * *The conclusions are need to be rewritten to highlight the key contributions of this manuscript*

*.*

Is the work clearly and accurately presented and does it cite the current literature?

Yes

If applicable, is the statistical analysis and its interpretation appropriate?

Yes

Are all the source data underlying the results available to ensure full reproducibility?

Yes

Is the study design appropriate and is the work technically sound?

Yes

Are the conclusions drawn adequately supported by the results?

Partly

Are sufficient details of methods and analysis provided to allow replication by others?

Yes

Reviewer Expertise:

Clinical Biochemistry

I confirm that I have read this submission and believe that I have an appropriate level of expertise to confirm that it is of an acceptable scientific standard.

# Response

**Published:** 2025-11-22

**Authors:** Ibe Michael Usman

**Affiliations:** Human Anatomy, Kampala International University - Western Campus, Bushenyi, Western Region, Uganda

**Reviewer Comment **I - This discussion could be expanded to explain the Ameliorative Effects of Ethyl-Acetate Extract of *Bidens pilosa* on Oxidative Stress and Reproductive Impairment

**Author’s Response:** The discussion is now expanded and mechanism of action for *Bidens pilosa* involving direct antioxidant activity has now been integrated with reproductive impairment.

**Reviewer Comment **I - The conclusions are need to be rewritten to highlight the key contributions of this manuscript

**Author’s Response: **The conclusion section has been rewritten and the contributions of the study have been stated in the manuscript.

# Reviewer report: Reviewer response for version 1

**DOI:** 10.5256/f1000research.183308.r410187

**Published:** 2025-09-09

**Authors:** Emmanuel Eimiomodebheki Odion

**Affiliations:** 1 University of Benin, Benin City, Edo, Nigeria

The phyto-compounds used for docking in this study were based on the threshold of 2 % percentage area (GC/MS analysis result) excluding many other compounds reported previously (Xuan and Khanh, 2016;Chemistry and pharmacology of *Bidens pilosa*: an overview) and considering that this same method of analysis is limited to compounds that are volatile in nature (meaning that non-volatile compounds are not captured). Suggestion: why not dock with all the Phyto-compounds from the GC/MS analysis and from that limit the subsequent protocols to the compounds that show preliminary activity.

**Others**

- Provide the authority for the plant and family at first mention.
- how long was the plant material dried?
- Suspension of fine powder in 400 mL of water, was for how long?
- Partitioning of the aqueous extract in different solvent three-times does not guaranty exhaustive extraction
- DPPH assay: The first 6 sentences are not part of the methodology
- Statistical analysis: the sentence should be recast to make sense.
- Not quite clear why the study was limited to Ethyl acetate fraction following initial partitioning into different solvent system.
- Was the crude EXTRACT used for the GC/MS?

Is the work clearly and accurately presented and does it cite the current literature?

Yes

If applicable, is the statistical analysis and its interpretation appropriate?

Yes

Are all the source data underlying the results available to ensure full reproducibility?

Partly

Is the study design appropriate and is the work technically sound?

Partly

Are the conclusions drawn adequately supported by the results?

Yes

Are sufficient details of methods and analysis provided to allow replication by others?

Partly

Reviewer Expertise:

Medicinal Chemistry

I confirm that I have read this submission and believe that I have an appropriate level of expertise to confirm that it is of an acceptable scientific standard, however I have significant reservations, as outlined above.

# Response

**Published:** 2025-11-22

**Authors:** Ibe Michael Usman

**Affiliations:** Human Anatomy, Kampala International University - Western Campus, Bushenyi, Western Region, Uganda

**Reviewer Comment** **II** -The phyto-compounds used for docking in this study were based on the threshold of 2 % percentage area (GC/MS analysis result) excluding many other compounds reported previously (Xuan and Khanh, 2016; Chemistry and pharmacology of *Bidens pilosa*: an overview) and considering that this same method of analysis is limited to compounds that are volatile in nature (meaning that non-volatile compounds are not captured). Suggestion: why not dock with all the Phyto-compounds from the GC/MS analysis and from that limit the subsequent protocols to the compounds that show preliminary activity.

**Author’s response** –We appreciate your comment and acknowledge the limitation of our GC/MS analysis method, which may have excluded some potentially active compounds. However, given the scope and objectives of our current study, we focused on the most abundant compounds (those above the 2% threshold) to prioritize computational resources and streamline our analysis. While docking all phyto-compounds from the GC/MS analysis is an excellent approach, it would significantly expand the computational workload, which wasn't feasible within our current project's constraints. Our goal was to identify potential leads from the most prominent compounds, recognizing that further studies can explore a broader range of compounds, including non-volatile ones, in the future.

**Others**

**Reviewer Comment** **II** – Provide the authority for the plant and family at first mention.

**Author’s response** – The authority for the plant and family have been captured at first mention

**Reviewer Comment** **II** – How long was the plant material dried?

**Author’s response** – The plant material was dried for 5 days

**Reviewer Comment** **II** – Suspension of fine powder in 400 mL of water, was for how long?

**Author’s response** - Suspension of fine powder in 400 mL of water, was for 48 hours

**Reviewer Comment** **II** – Partitioning of the aqueous extract in different solvent three-times does not guaranty exhaustive extraction

**Author’s response** – This is well noted

**Reviewer Comment** **II** – DPPH assay: The first 6 sentences are not part of the methodology

**Author’s response** – The first 6 sentences have been deleted

**Reviewer Comment** **II** – Statistical analysis: the sentence should be recast to make sense.

**Author’s response** - The sentence have been rewritten to make sense

**Reviewer Comment** **II** – Not quite clear why the study was limited to Ethyl acetate fraction following initial partitioning into different solvent system.

**Author’s response** – The preference for Ethyl acetate fraction was due to its oil-like composition.

**Reviewer Comment** **II** – Was the crude EXTRACT used for the GC/MS?

**Author’s response** – The crude extract was not used for GC/MS due to financial constrain at the time study was conducted
